# The effects of high plasma levels of Aβ_1-42_ on mononuclear macrophage in mouse models of Alzheimer’s disease

**DOI:** 10.1186/s12979-023-00366-4

**Published:** 2023-07-31

**Authors:** Chunrong Li, Kangding Liu, Jie Zhu, Feiqi Zhu

**Affiliations:** 1 Department of Neurology, Neuroscience Center, The First Hospital of Jilin University, Jilin University, Changchun, 130021 China; 2 Cognitive Impairment Ward of Neurology Department, The Third Affiliated Hospital of Shenzhen University, Shenzhen, 518055 China; 3 Department of Neurobiology, Care Sciences & Society, Division of Neurogeriatrics, Karolinska Institutet, Karolinska University Hospital Solna, Stockholm, Sweden

**Keywords:** Alzheimer’s disease, Aβ_1-42_, Mononuclear macrophage, Myeloid derived suppressor cells, Bone marrow myeloid progenitor cells

## Abstract

More and more evidences are proving that microglia play a crucial role in the pathogenesis of Alzheimer’s disease (AD) and the plasma Aβ_1-42_ levels significantly increased 15 years before the onset of dominantly inherited AD. However, the effects of high plasma levels of Aβ_1-42_ on mononuclear macrophage, the peripheral counterparts of microglia, remain unclear. In the present study, we used APP/PS1 transgenic (Tg) mice and a parabiotic model of wild type (Wt) mice and Tg mice (Parabiotic Wt-Tg, Pa (Wt-Tg)) to investigate the effects of high plasma levels of Aβ_1-42_ on peripheral mononuclear macrophage. Our results showed that in the early stage of Tg mice (7 months) and Pa (Wt-Tg) mice (4 months), the proportions of pro-inflammatory macrophages in peritoneal cavity, myeloid derived suppressor cells (MDSCs) in spleen, granulocyte-monocyte progenitors (GMPs) in bone marrow, and the plasma levels of interleukin-6 (IL-6) were significantly decreased. While the proportions of pro-inflammatory macrophages, MDSCs, GMPs, and the plasma levels of IL-6 and tumor necrosis factor (TNF)-α, as well as the numbers of bone marrow-derived macrophages (BMDMs) in mice brain were increased in the late stage of Tg mice (11 months) and Pa (Wt-Tg) mice (8 months). In addition, the proportions of monocytes in spleen and the proliferation of bone marrow cells (BMCs) were enhanced consistently, and the phagocytic function of macrophages kept stably after high plasma levels of Aβ_1-42_ sustaining stimulation. These results demonstrated that high plasma levels of Aβ_1-42_ play a biphasic regulating role at different stages of the disease, namely inhibiting effects on peripheral pro-inflammatory macrophages in the early stage of AD model, while promoting effects in the late stage of AD model. The mechanism behind this may be associated with their effects on MDSCs in spleen and myeloid progenitor cells in bone marrow. Therefore, intervening the effects of plasma Aβ_1-42_ on pro-inflammatory macrophages might offer a new therapeutic approach to AD.

## Introduction

Alzheimer’s disease (AD), the most common neurodegenerative disorder with progressive memory and cognitive loss, is affecting almost 50 million people worldwide, and the incidence of AD is increasing rapidly with the ageing of the world population [1]. The medical care and nursing cost of AD is enormous [2–4]. The main neuropathological features of AD are neuritic plaques generated by extracellular β-amyloid (Aβ) deposition, neurofibrillary tangles formed by intracellular accumulation of hyperphosphorylated tau (p-Tau), and neuroinflammation, neuron and synapse loss, as well as astroglial proliferation [5]. Major pathogenic hypotheses of AD focus on the Aβ cascade and p-Tau accumulation. However, clinical treatment trials including designed to test the effects of inhibiting Aβ_1-42_ production by β-secretase inhibitor, clearing Aβ_1-42_ by monoclonal antibodies, and inhibiting p-Tau by leuco-methylthioninium bis (hydromethanesulfonate; LMTM), have all failed to demonstrate clinical efficiency [1, 6, 7]. Thus, the hypotheses of Aβ cascade and p-Tau have been challenged [8–11]. Accumulating studies suggest that neuroinflammation plays a key role in the pathogenesis of AD [12]. Microglia, the main resident immune cells in the central nervous system (CNS), act as vigilant housekeepers in the adult brain; they activate immediately and switch their behavior from patrolling to shielding the injured site when the blood–brain barrier (BBB) is disrupted [13]. In the pathogenesis of AD, microglia might have a double-edged sword role. At the early stage of AD, microglia protect brain from the toxic effects of Aβ_1-42_ by phagocytizing and clearing Aβ. While, with AD progresses, microglia lose clearing Aβ ability and produce pro-inflammatory cytokines and molecules that promote Aβ deposition [14]. Genome-wide analysis suggests that several genes, encoding for glial clearance of Aβ and inflammatory reaction, increase the risk of sporadic AD [15, 16]. In addition, complement and microglia mediate the early loss of synapses in AD mouse models [17]. By the vivo PET images, microglial activation was found to be correlated with both tau and amyloid [18], moreover, the co-occurrence of Aβ, tau and microglia activation was the strongest predictor of cognitive impairment [19].

Traditionally, brain has been regarded as an immune-privileged organ protected by the BBB. But recently, accumulating evidence shows that there are interactions between brain and peripheral organs, having a significant role in the development and progression of AD [20]. In the late stage of AD, with the disruption of BBB, peripheral immune cells and inflammatory molecules entered into brain parenchyma [21]. The role of peripheral innate immunity in the pathogenesis of AD has gained more attentions in recent years [22, 23].

Several studies have found that circulating bone marrow-derived macrophages (BMDMs) can enter into brain tissue, where they serve as bone marrow-derived microglia and have more efficiently phagocytic ability of Aβ_1-40/1–42_ compared to resident microglial cells [24, 25]. Selective ablation of bone marrow-derived dendritic cells increases amyloid plaques in AD mouse models [26]. Increased cerebral infiltration of monocytes, either by elevating the levels of circulating monocytes or by weekly treatment with glatiramer acetate (which simulates myelin basic protein), substantially attenuated disease progression in AD mouse models [27]. Indeed, prior to these observations, monocytic cells derived from bone marrow stem cells had been used to treat AD [28], and long-term use of nonsteroidal anti-inflammatory drugs (NSAIDs) prior to the onset of AD offered protection against AD [29–31]. In November of 2019, sodium oligomannate (GV-971), a marine algae-derived oral oligosaccharide, was approved for AD treatment by the Chinese Food and Drug Administration based on its ability to alleviate neuroinflammation by regulating gut microbiota and inhibiting the brain infiltration of peripheral T helper (Th) 1 cells [32]. There is evidence that circulating neutrophils can extravasate and surround Aβ deposits, where they secrete interleukin-17 (IL-17) and neutrophil extracellular traps (NETs). Moreover, inhibiting neutrophil trafficking or depleting these cells reduces AD-like neuropathological changes and improves memory of AD mice [33]. So, motivating monocytic cells derived from bone marrow stem cells had been regarded as a promising therapy in AD [28].

In sporadic AD patients, the levels of plasma Aβ_1-42_ were decreased significantly [34], however, plasma Aβ_1-42_ levels were increased significantly 15 years in dominantly inherited AD and Down syndrome before the onset of symptoms compared with normal people [35, 36]. As well as Aβ_1-42_ levels in plasma neuronal-derived exosome (NDE) of mild cognitive impairment (MCI) and AD were significantly higher than those in normal controls [37]. Moreover, in the wild-type (Wt) mice parabiosis with APPswe/PS1dE9 (APP/PS1) transgenic (Tg) mice or transplanting bone marrow cells (BMCs) from APP/PS1 Tg mice, the plasma Aβ_1-42_ levels were significantly increased and plasma Aβ_1-42_ entered into Wt mice brain to form cerebral amyloid angiopathy (CAA) and Aβ plaques similar to Tg mice [38, 39]. Until now, the effects of high plasma Aβ_1-42_ levels on mononuclear macrophage, the peripheral counterparts of microglia, remain unclear. The present study employed parabiosis model of AD to investigate the effects of high plasma levels of Aβ_1-42_ on peripheral mononuclear macrophage in order to explore the underlying mechanisms and look for the new possible therapeutical target for AD.

## Materials and methods

### Parabiosis

**Fig. 1:**
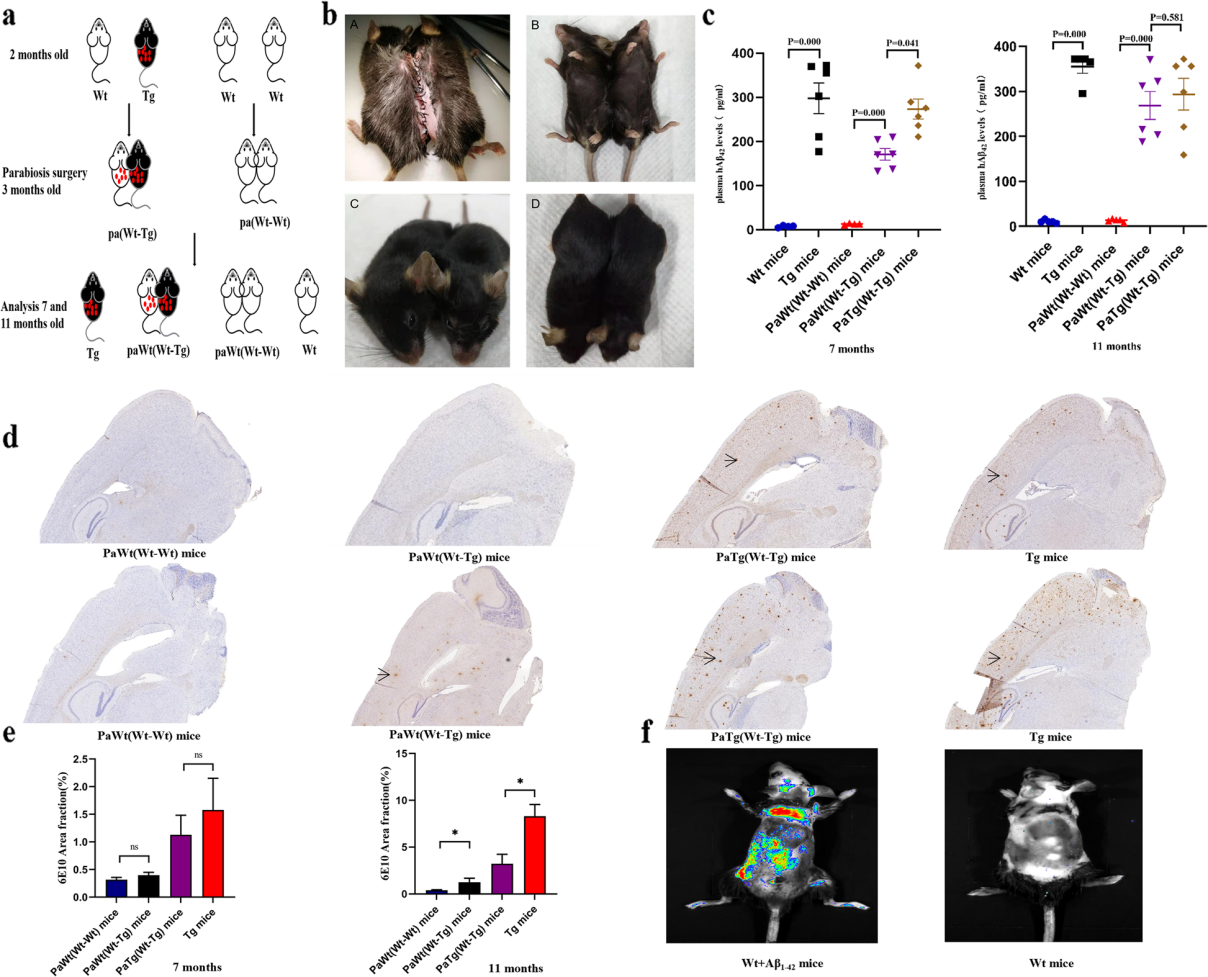
The plasma levels of Aβ_1-42_ and brain deposition of Aβ after parabiotic surgery. **a** Schematic diagram depicting the parabiotic pairings. A pair of 3 months-old female Tg mice and age-matched female Wt littermates were used for parabiosis. A pair of 3 months-old female Wt mice were used for parabiotic controls. Samples were collected for analysis at 7 and 11 months of age. Age-matched female Tg and Wt mice without parabiosis were also explored. **b** The mice after parabiotic surgery. **c** Plasma Aβ_42_ levels in Wt, PaWt(Wt-Wt), PaWt(Wt-Tg), PaTg(Wt-Tg), and Tg mice at the age of 7 and 11 months. At the age of 7 months, the plasma Aβ_42_ levels in Tg mice were significantly higher than Wt mice (*p* = 0.000), the plasma Aβ_42_ levels in PaWt(Wt-Tg) mice were significantly higher than PaWt(Wt-Wt) mice (*p* = 0.000), the plasma Aβ_42_ levels in PaTg(Wt-Tg) mice were higher than PaWt(Wt-Tg) mice (*p* = 0.041). At the age of 11 months, the plasma Aβ_42_ levels in Tg mice were significantly higher than Wt mice (*p* = 0.000), the plasma Aβ_42_ levels in PaWt(Wt-Tg) mice were significantly higher than PaWt(Wt-Wt) mice (*p* = 0.000), the plasma Aβ_42_ levels in PaTg(Wt-Tg) mice were higher than PaWt(Wt-Tg) mice (*p* = 0.581). **d** Representative images of amyloid deposition stained with 6E10 in neocortex and hippocampus of PaWt(Wt-Wt), PaWt(Wt-Tg), PaTg(Wt-Tg), and Tg mice brain at the age of 7 and 11 months respectively. Scale bars 2500 µm. **e** The quantifications of the Aβ plaques (6E10Area fraction (%)) in the neocortex and hippocampus were 1.58 ± 0.57% in Tg mice, 1.13 ± 0.35% in PaTg(Wt-Tg) mice, 0.40 ± 0.05% in PaWt(Wt-Tg) mice, and 0.32 ± 0.04% in PaWt(Wt-Wt) mice, respectively at the age of 7 months. At the age of 11 months the quantifications of the Aβ plaques in the neocortex and hippocampus were 8.13 ± 1.25% in Tg mice,3.03 ± 0.88% in PaTg(Wt-Tg) mice, 1.24 ± 0.44% in PaWt(Wt-Tg) mice, and 0.40 ± 0.06% in PaWt(Wt-Wt) mice, respectively.At the age of 7 months, the Aβ plaques in the neocortex and hippocampus had no significant difference between PaWt(Wt-Tg) mice and PaWt(Wt-Wt) mice (*p* = 0.114), and Tg mice and PaTg(Wt-Tg) mice (*p* = 0.200). The Aβ plaques in the neocortex and hippocampus of PaWt(Wt-Tg) were higher compared with PaWt(Wt-Wt) (*p* = 0.029), and in Tg mice the Aβ plaques in the neocortex and hippocampus were higher compared with PaTg(Wt-Tg) mice (*p* = 0.029). **f** Bioluminescent images of Wt mice after tail intravenous injection with hAβ_42_ labeled with HiLyte Fluor 488 1 h. (*n* = 6, Mean ± SD, one-way analysis of variance. ***P < 0.001, **P < 0.01, ns denotes no statistical significance)

All experiments involving mice were approved by the Laboratory Animal Welfare and Ethics Committee of Shenzhen Luohu Hospital Group (SLHG), Shenzhen, China. To exclude the influence of gender on brain Aβ deposition, 6–8 weeks old female pathogen-free APP/PS1 mice and C57BL/6 Wt mice were used in the present study. All mice were purchased from Shanghai Model Organisms Center, Inc (SMOC, Shanghai, China). Animals acclimatized for 2–5 days in the pathogen-free animal facility of the SLHG Precision Medicine Research Institute. Mice were housed in a room with a 12- hour light–dark cycle and provided with food and water at liberty. Parabiosis surgery was performed on pairs of mice after they had adapted to each other by living together in a cage for 1 month at the age of 3 months according to the procedure from a previous study [40] (Fig. 1a). Firstly, ketamine (100 mg/kg), xylazine (20 mg/kg), and acepromazine (3 mg/kg) were used to anesthetize animals, then placed animals in a parallel orientation. A left lateral incision was made on one mouse while a right one was made on the partner mouse (from the back of the ear to the back of the thigh). The head end and tail end are threaded using 5–0 nylon without sutures respectively. The scapulae and femoral bone of the mice were fixed together with 4–0 silk sutures. Then, the corresponding dorsal and ventral skin was sutured with 4–0 silk. After the surgery, before transferred into the husbandry area, the parabiotic mice were allowed to recover in a warm and clean environment (Fig. 1b). Prophylactic antibiotic treatment (enrofloxacin, 5 mg/kg) was started 1 day prior to the surgery and continued for 2 weeks.

Parabiosis was maintained for 4 and 8 months, respectively, while age and weight-matched female Wt and Tg mice (*n* = 6, for each group) without parabiosis were used as controls. In order to investigate the effects of short-term stimulation of high plasma Aβ_1-42_ levels on mononuclear macrophage, exogenous Aβ_1-42_ peptide labeled with HiLyte Fluor 488 (AnaSpec, Fremont, CA, USA) 200ul of 100uM in phosphate buffer saline (PBS) was intravenously injected into 6–8 weeks-old female Wt mice via the tail vein (*n* = 6) for three times in a week, and the age and weight matched female Wt mice (*n* = 6) were used as controls. The mice in control group received the same volume of vehicle. Bioluminescent imaging was performed using the IVIS spectrum imaging system (Caliper Life-Sciences, Hopkinton, MA, USA) 1 h (h) after tail intravenous injection (Fig. 1f).

### Cell preparation

Parabiotic animals were sacrificed at 7 and 11 months old, respectively, namely after 4 and 8 months sustaining stimulation with high plasma levels of Aβ_1-42_, and 6–8 weeks-old Wt mice after tail vein with Aβ_1-42_ peptide one week were also sacrificed. The monocytes and myeloid derived suppressor cells (MDSCs) suspensions were obtained from mice spleen by pressing them through a 70 um Falcon cell strainer, macrophages of abdominal cavity were harvested by washing three times with 10 ml of RPMI 1640 medium, and BMCs were isolated by flushing the marrow from mice femoral and tibial bone. All the above cell suspensions were collected and rinsed, centrifuged for 10 min (mins) with RPMI 1640. After red blood cells lysis, adjusted the cells concentration to 2 × 10^6^/ml by using complete medium with penicillin and streptomycin (1%; Hyclone, Logan, UT) and fetal bovine serum (10%; Sigma Aldrich, St Louis, MO), prepared for the following experiments.

### Measurement of the proportions of mononuclear macrophage, MDSCs, and BMCs by flow cytometry

The monocytes, macrophages, pro- and anti-inflammatory macrophages, MDSCs, and BMCs were incubated in 0.5ug Fc Block (cat. 51–9006315, BD Biosciences) for 15 min at room temperature to block nonspecific responses. All staining was performed in a dark environment at 4℃. Monocytes and MDSCs from mice spleen were incubated with the following antibodies: BB515-conjugated anti-mouse CD11b (cat. 564454, BD Pharmingen TM), PerCP-Cy5.5-conjugated anti-mouse CD115 (cat. 745906, BD Pharmingen TM), PE-conjugated anti-mouse Ly6G (cat. 560599, BD Pharmingen TM), and APC-conjugated anti-mouse Ly6C (cat. 560592, BD Pharmingen TM) for 30 min. Each monocyte and MDSC sample were stained in three duplicate tubes.

Macrophages from abdominal cavity were incubated with antibodies BB515-conjugated anti-mouse CD11b (cat. 564454, BD Pharmingen TM), BB700-conjugated anti-mouse F4/80 (cat.746070, BD Pharmingen TM), BV510-conjugated anti-mouse CD40 (cat. 745041, BD Pharmingen TM) for 30 min at 4 °C to detect extracellular molecules. Then cells were permeabilized with the Fixation/Permeabilization solution Kit (cat. 554714, BD Pharmingen TM) for 20 min. The intracellular molecules were stained with antibodies BV421-conjugated anti-mouse CD206 (cat. 141717, BioLegend), APC-conjugated anti-mouse arginase-1 (Arg-1) (cat. 5868A, R&D systems), and PE-conjugated anti-mouse inducible nitric oxide synthase (iNOS) (cat. 12–5929-82, Invitrogen) for 30 min at 4 °C. Flow cytometric analysis was performed by using FLOWJO 7.6.1 (Treestar).

BMCs were stained with the BD Stemflow TM mouse Hematopoietic Stem Cell (HSC) Isolation Kit (cat. 560492, BD Pharmingen TM) which containing 7-amino-actinomycin D, FITC-conjugated anti-mouse CD34, PE-conjugated anti-mouse c-Kit, PE-Cy7-conjugated anti-mouse Sca-1, and APC-conjugated anti-mouse Lineage antibody Cocktail. BV421-conjugated anti-mouse CD135 (cat. 562898, BD Pharmingen TM) and BV510-conjugated anti-mouse CD16/32 (cat. 740111, BD Pharmingen TM) were also used to stain BMCs. All the antibodies were incubated for 1 h (h) at 4℃.

### Analysis of cytokines and Aβ_1-42_ levels

The cytokines levels of mice plasma, including IL-6 (cat. 558301), IL-12p70 (cat. 558303), TNF-α (cat. 558299), IL-10 (cat. 558300), and IL-1β (cat. 560232) were evaluated by using Cytometric Beads Array (CBA) Flex Set (BD-Pharmingen, La Jolla, CA, USA). Briefly, mice soluble protein flex set capture beads were mixed and washed with 0.5ml wash buffer and centrifuged at 200g for 5min. The supernatant was carefully discarded by aspiration without disturbing the bead pellet. The beads were then resuspended in capture bead diluent to a final concentration of 50ul/test and incubated for 15min at room temperature. Afterward, 50ul capture beads were mixed with 50ul sample or standard and incubated at room temperature for 1h. Subsequently, 50ul mixed phycoerythrin (PE) detection reagent was added to each assay tube and incubated at room temperature for 2h. Next, 1ml wash buffer was added to each assay tube and centrifuged at 270g for 5min. After carefully aspirating and discarding the supernatant from each assay tube, 300ul buffer was added to each assay tube to resuspend the beads. Samples were analyzed by flow cytometry (excitation wavelength 565nm and emission wavelength 578nm) and the data was acquired. Data were analyzed using FCAP Array software.

The plasma levels of Aβ_1-42_ (cat. CEA947Hu, Cloud-Clone Corp, Wuhan, China) and inflammatory cytokines transforming growth factor-β (TGF-β) (cat. SEA124Mu, Cloud-Clone Corp, Wuhan, China) were measured by enzyme-linked immunosorbent assay (ELISA) kit according to the manufacturer’s instructions. The minimum detectable dose of hAβ_42_ kit was typically 1.44 pg/ml and the minimum detectable dose of TGF-β kit was typically 15.6 pg/ml. The plasma hAβ_42_ levels of Tg mice and PaWt(Wt-Tg) mice were run with 1:2 serial dilutions.

### Assay of the phagocytic function of peritoneal macrophage

1 × 10^5^ macrophages collected from abdominal cavity were plated in 24-well plates incubated with HiLyte Fluor 488-labeled Aβ_1-42_ (cat. AS-60479, AnaSpec, Fremont, CA, USA) 1ug/ml for 24 h. After incubation, the macrophages were washed with phosphate buffer saline (PBS) to remove excess Aβ_1-42_ and stained with APC-Cy7-conjugated anti-mouse CD11b (cat. 557,657, BD Pharmingen TM) and BB700-conjugated anti-mouse F4/80 (cat. 746,070, BD Pharmingen TM) for 30 min at 4℃. Flow cytometric analysis was performed by using FLOWJO 7.6.1 (Treestar).

### Analysis of proliferative function of BMC

BMC proliferation was measured by CCK-8 kits (cat. FC101, TransDetect, Beijing, China). A total of 1 × 10^4^ cells in a volume of 100ul per well were cultured in a 96-well plate medium containing 10% fetal bovine serum (FBS) for 2, 5, 6, and 7 days respectively and each sample had three replicate wells. The CCK-8 reagent (10ul) was added to 90ul RPMI 1640 to generate a working solution, of which 100ul was added per well and incubated for 4 h (hs). The optical density (OD) was measured at 450 nm.

### Detection of Aβ deposition and bone marrow-derived macrophages (BMDMs) in mice brain using immunohistochemical and immunofluorescence assays

The brain slices were deparaffinized and dehydrated with xylene and ethanol. Tissues were then incubated in 3% H_2_O_2_ for 5–10 min to eliminate endogenous peroxidase activity. The tissues were incubated with primary antibody at a working solution and incubated at 4 ℃ overnight. We used 6E10 (cat. 803,007, biolgend) to label Aβ and IBA-1^+^ (cat. 019–19741, WAKO), CD68^+^ (cat. MCA1957T, biorad), P2Y12^−^ (cat. NBP2-33,870, novus) to label BMDMs. After blocking in PBS containing 30% bovine serum albumin (BSA) for 30min at room temperature. Next HRP conjugated secondary antibody was added and incubated for 2hs at room temperature. Finally, the samples were fixed with PBS containing 10% 4,6-diamidino-2-phenylindole (DAPI) for 2min. The results of the samples were observed using a confocal microscope (Zeiss LSM 800, Carl Zeiss Microimaging Inc., NY, USA) under a 20 X objective. 5 images were taken per mouse and Image J used to count the amount of BMDMs.

### Statistical analysis

Differences among groups were tested by the rank-sum test. Differences of categorical data were tested by Chi-square. For all statistical tests, two-sided *P*-values less than 0.05 were defined as statistically significant. All analyses were carried out using Statistical Package for Social Sciences (SPSS) version 23.0 software (IBM, West Grove, PA, USA).

## Results

### Increased the plasma levels of Aβ_1-42_ and brain deposition of Aβ in PaWt(Wt-Tg) mice after parabiotic surgery

The parabiotic animal models of Wt mice and Tg mice (parabiotic Wt-Tg, Pa(Wt-Tg)), Wt mice and Wt mice (parabiotic Wt-Wt, Pa(Wt-Wt)) were established. We measured the Aβ_1-42_ plasma levels of Wt mice, Tg mice, Wt mice of Pa(Wt-Wt) (PaWt(Wt-Wt)) mice, Wt mice of Pa(Wt-Tg) (PaWt(Wt-Tg)) mice, and Tg mice of Pa(Wt-Tg) (PaTg(Wt-Tg)) mice, respectively.

We found after parabiosis from 3 months of age to 7 and 11 months of age, respectively, the Aβ_1-42_ plasma levels of PaWt(Wt-Tg) mice were significantly increased than PaWt(Wt-Wt) mice (at the age of 7 and 11 months *p* = 0.000) (Fig. 1c). We used 6E10 to label Aβ, as shown in the Fig. 1d, both at the age of 7 and 11 months, Aβ plaques were found in the neocortex and hippocampus of Tg mice. After parabiosis for 4 months, Aβ plaques were scarce in PaWt(Wt-Tg) mice brain, but after 8 month, they were obvious presented in neocortex and hippocampus of PaWt(Wt-Tg) mice brain (Fig. 1e).

As shown in Fig. 1c-e, these results showed that Aβ_1-42_ in plasma of Tg mice entered into the Wt mice blood circulation and then deposited in the cerebral vascular wall and brain parenchyma of Wt mice after parabiotic surgery, demonstrating that the PaTg(Wt-Tg) mice can stimulate persistently Wt mice through providing stable high levels of Aβ_1-42_ leading to Aβ deposition in brain of PaWt(Wt-Tg) mice.

### Enhanced infiltrations of BMDMs in brain of PaWt(Wt-Tg) mice after parabiotic surgery

**Fig. 2:**
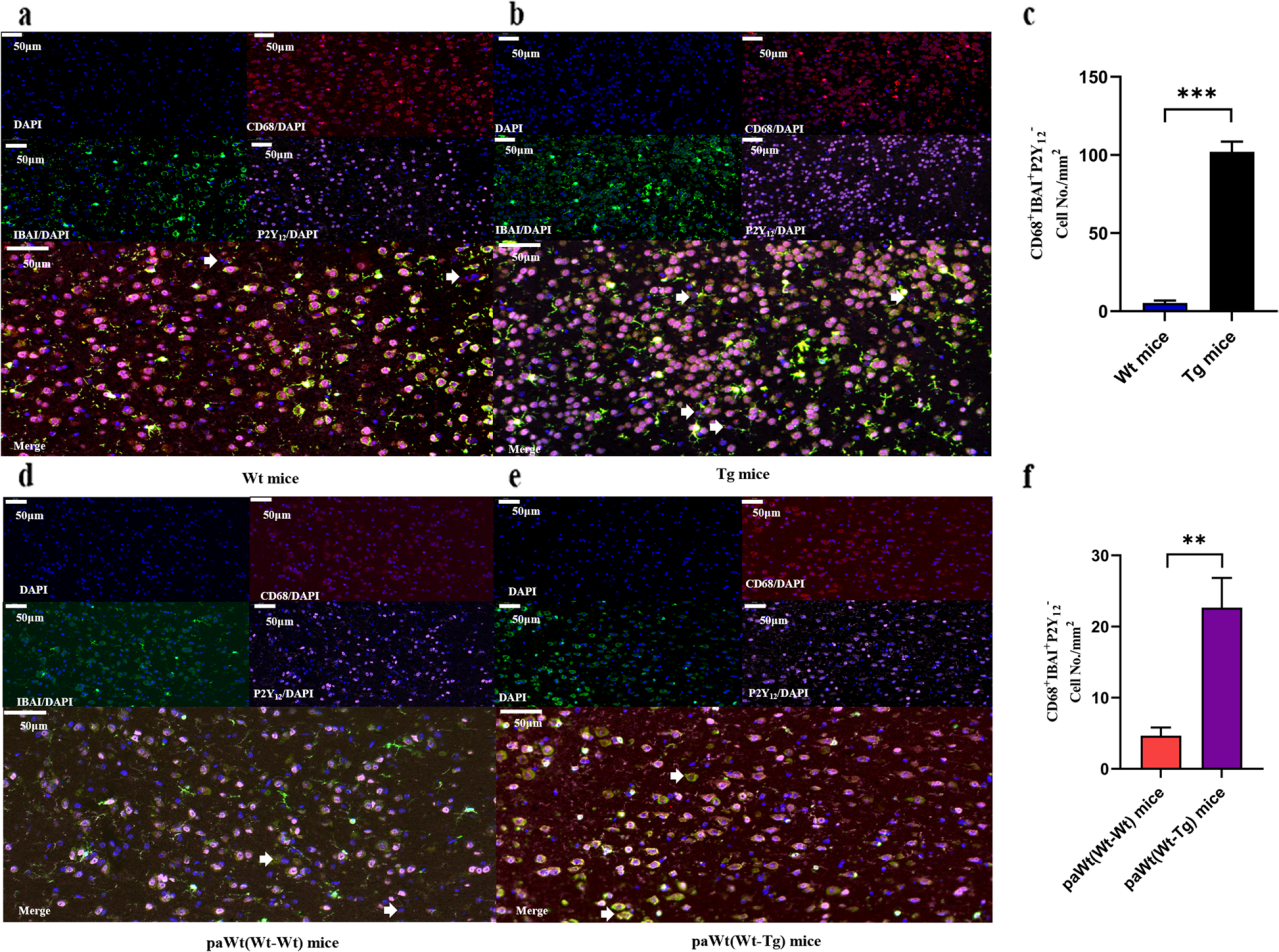
The infiltrations of BMDMs in brain after parabiotic surgery. **a** Immunofluorescence image of microglia and BMDMs co-stained with CD68, IBAI, and P2Y_12_ antibodies in neocortex and hippocampus of Wt mice brain at the age of 11 months. Scale bars: 500 µm. **b** Immunofluorescence image of microglia and BMDMs co-stained with CD68, IBAI, and P2Y_12_ antibodies in neocortex and hippocampus of Tg mice brain at the age of 11 months. Scale bars: 500 µm. **c** The statistical diagram of the amounts of BMDMs in neocortex and hippocampus of Wt and Tg mice brain at the age of 11 months. The amounts of BMDMs in neocortex and hippocampus of Tg mice were significant higher compared with Wt mice at the age of 11 months (*p* = 0.000). **d** Immunofluorescence image of microglia and BMDMs co-stained with CD68, IBAI, and P2Y_12_ antibodies in neocortex and hippocampus of PaWt(Wt-Wt) mice brain at the age of 11 months. Scale bars: 500 µm. **e** Immunofluorescence image of microglia and BMDMs co-stained with CD68, IBAI, and P2Y_12_ antibodies in neocortex and hippocampus of PaWt(Wt-Tg) mice brain at the age of 11 months. Scale bars: 500 µm. **f** The statistical diagram of the amounts of BMDMs in neocortex and hippocampus of PaWt(Wt-Wt) and PaWt(Wt-Tg) mice brain at the age of 11 months. The amounts of BMDMs in neocortex and hippocampus of PaWt(Wt-Tg) mice brain were significantly higher than PaWt(Wt-Wt) mice at the age of 11 months (*p* = 0.004). 5 images were taken per mouse and Image J used to count the amounts of BMDMs. (*n* = 6, Scale bars 500 µm. ***P < 0.001, **P < 0.01, ns denotes no statistical significance)

Detection of IBA-1^+^CD68^+^P2Y12^−^ cells in the neocortex and hippocampus represented infiltrations of BMDMs in brain. At the age of 7 months there was no difference between Tg mice and Wt mice brains regarding the infiltrating amounts of IBA-1^+^CD68^+^ P2Y12^−^ cells. Similarly, it was also no difference of these cells infiltration in brain between PaWt(Wt-Tg) mice and PaWt(Wt-Wt) mice after parabiosis for 4 months (All data not shown).While at the age of 11 months, the amounts of these cells in Tg mice brain were higher than in Wt mice (*p* = 0.000) (Fig. 2a-c). Interestedly, the amounts of these cells in PaWt(Wt-Tg) mice brain were significant enhanced compared with PaWt(Wt-Wt) mice after parabiosis for 8 months (*p* = 0.004) (Fig. 2d-f).

### A biphasic regulating effect of the high plasma Aβ_1-42_ on proportions of mononuclear macrophage in PaWt(Wt-Tg) mice

The proportions of mononuclear macrophage between Tg mice and Wt mice at the age of 7 and 11 months, and PaWt(Wt-Tg) mice and PaWt(Wt-Wt) mice after parabiosis for 4 and 8 months were compared respectively in our study. In PaWt(Wt-Wt) mice, we collected the data from both Wt mice in the parabiosis.

**Fig. 3:**
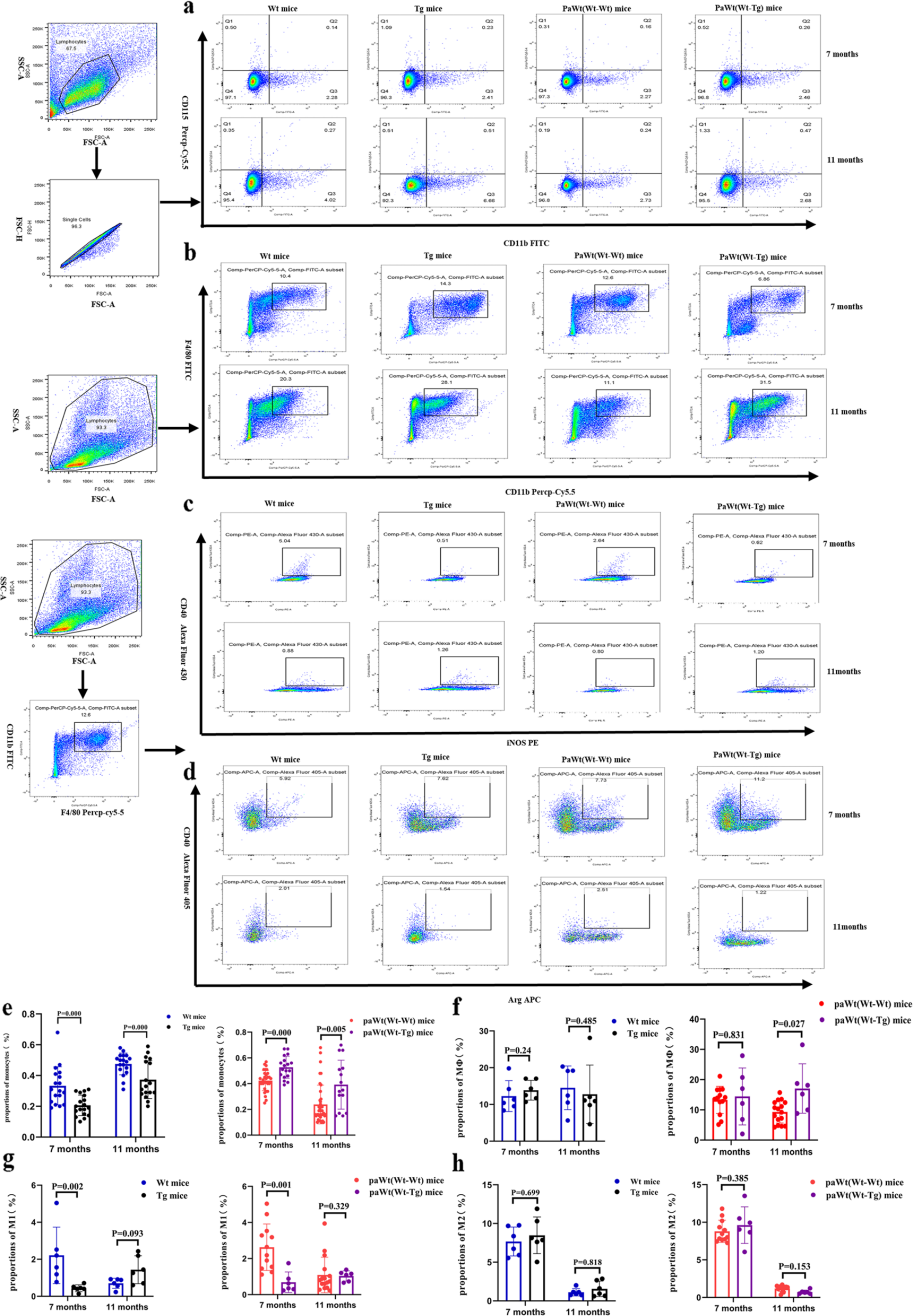
The effects of high plasma Aβ_1-42_ on proportions changes of mononuclear macrophage. **a** The alterations of monocytes proportions in Wt, Tg, PaWt(Wt-Wt), and PaWt(Wt-Tg) mice spleen MNCs. Monocytes were stained with CD11b^+^CD115^+^. **b** The alterations of macrophages proportions in Wt, Tg, PaWt(Wt-Wt), and PaWt(Wt-Tg) mice abdominal cavity MNCs. Macrophages were stained with CD11b^+^F4/80^+^. **c** The alterations of pro-inflammatory macrophages proportions in macrophages of Wt, Tg, PaWt(Wt-Wt), and PaWt(Wt-Tg) mice abdominal cavity. The pro-inflammatory macrophages were stained with CD40^+^iNOS^+^. **d** The alterations of anti-inflammatory macrophages proportions in macrophages of Wt, Tg, paWt(Wt-Wt), and paWt(Wt-Tg) mice abdominal cavity. The anti-inflammatory macrophages were stained with CD206^+^Arg-1.^+^. **e** The statistical diagram of monocytes proportions in Wt, Tg, PaWt(Wt-Wt), and PaWt(Wt-Tg) mice spleen MNCs. In Tg mice spleen mononuclear cells (MNCs) were significantly higher than Wt mice (at the age of 7 months *p* = 0.000, at the age of 11 months p = 0.000). After parabiosis for 4 and 8 months, the proportions of monocytes in PaWt(Wt-Tg) mice spleen MNCs were significantly higher than PaWt(Wt-Wt) mice (after parabiosis for 4 months *p* = 0.000, after parabiosis for 8 months *p* = 0.005). **f** The statistical diagram of macrophages proportions in Wt, Tg, PaWt(Wt-Wt), and PaWt(Wt-Tg) mice abdominal cavity MNCs. At the age of 7 and 11 months, the proportions of macrophages in Tg mice abdominal cavity MNCs had no significantly difference compared with Wt mice (at the age of 7 months *p* = 0.24, at the age of 11 months *p* = 0.485). The proportions of macrophages in abdominal cavity MNCs had no significantly difference in PaWt(Wt-Tg) mice compared with PaWt(Wt-Wt) mice after parabiosis for 4 and 8 months (after parabiosis for 4 months *p* = 0.831, after parabiosis for 8 months *p* = 0.027). **g** The statistical diagram of pro-inflammatory macrophages proportions in macrophages of Wt, Tg, PaWt(Wt-Wt), and PaWt(Wt-Tg) mice abdominal cavity. The proportions of pro-inflammatory macrophages in macrophages of Tg mice abdominal cavity MNCs were decreased significantly (*p* = 0.002) at the age of 7 months, and the proportions of pro-inflammatory macrophages in Tg mice abdominal cavity MNCs had no significantly difference compared with Wt mice at the age of 11 months (*p* = 0.093). The proportions of pro-inflammatory macrophages in macrophages of PaWt(Wt-Tg) mice abdominal cavity MNCs were decreased significantly after parabiosis for 4 months (*p* = 0.001), and the proportions of pro-macrophages had an increased tendency in PaWt(Wt-Tg) mice compared with PaWt(Wt-Wt) mice after parabiosis for 8 months (*p* = 0.329). **h** The statistical diagram of anti-inflammatory macrophages proportions in macrophages of Wt, Tg, paWt(Wt-Wt), and paWt(Wt-Tg) mice abdominal cavity. The proportions of anti-inflammatory macrophages in Tg mice abdominal cavity MNCs had an increased tendency compared with Wt mice at the age of 7 months (*p* = 0.699) and at the age of 11 months, the proportions of anti-inflammatory macrophage in Tg mice abdominal cavity MNCs had no significantly difference compared with Wt mice (*p* = 0.818). The proportions of anti-inflammatory macrophages in PaWt(Wt-Tg) mice abdominal cavity MNCs had an increased tendency compared with PaWt(Wt-Wt) mice after parabiosis for 4 months (*p* = 0.385), and after parabiosis for 8 months, the proportions of anti-inflammatory macrophages in PaWt(Wt-Tg) mice abdominal cavity MNCs had no significant alterations compared with PaWt(Wt-Wt) mice (*p* = 0.153). (Wt, Tg, and PaWt(Wt-Tg) mice *n* = 6; PaWt(Wt-Wt)mice *n* = 12, data from the two Wt mice of Pa(Wt-Wt) mice, Mean ± SD, one-way analysis of variance. ***P < 0.001, **P < 0.01, *P < 0.05, ns denotes no statistical significance)

As shown in Fig. 3a, at the ages of 7 and 11 months, the proportions of monocytes (both CD11b and CD115 positive) in Tg mice spleen mononuclear cells (MNCs) were significantly higher than Wt mice (at the age of 7 months *p* = 0.000, at the age of 11 months *p* = 0.000) (Fig. 3a, e). Consistently, after parabiosis for 4 and 8 months, the proportions of monocytes in PaWt(Wt-Tg) mice spleen MNCs were significantly higher than PaWt(Wt-Wt) mice (after parabiosis for 4 months *p* = 0.000, after parabiosis for 8 months *p* = 0.005) (Fig. 3a, e).

At the age of 7 months, the proportions of macrophages (both CD11b and F4/80 positive) in Tg mice abdominal cavity MNCs had no significantly difference compared with Wt mice (*p* = 0.24) (Fig. 3b, f). The proportions of pro-inflammatory macrophages (both CD40 and iNOS positive) in macrophages of Tg mice abdominal cavity MNCs were decreased significantly (*p* = 0.002) and the proportions of anti-inflammatory macrophages (both Arg-1 and CD206 positive) had an increased tendency compared with Wt mice (*p* = 0.699) (Fig. 3c-d, g-h). At the age of 11 months, the proportions of macrophages (*p* = 0.485), pro-inflammatory macrophages (*p* = 0.093), and anti-inflammatory macrophage (*p* = 0.818) in Tg mice abdominal cavity MNCs had no significantly difference compared with Wt mice (Fig. 3c-d, g-h). Furthermore, the proportions of macrophages in abdominal cavity MNCs had no significantly difference in PaWt(Wt-Tg) mice compared with PaWt(Wt-Wt) mice after parabiosis for 4 months (*p* = 0.831) (Fig. 3b, f). In addition, the proportions of pro-inflammatory macrophages in macrophages of PaWt(Wt-Tg) mice abdominal cavity MNCs were decreased significantly (*p* = 0.001) and anti-inflammatory macrophages had an increased tendency compared with PaWt(Wt-Wt) mice after parabiosis for 4 months (*p* = 0.385) (Fig. 3c-d, g-h). The proportions of macrophages in PaWt(Wt-Tg) mice abdominal cavity MNCs were increased significantly compared with PaWt(Wt-Wt) mice after parabiosis for 8 months (*p* = 0.027). However, the proportions of anti-inflammatory macrophages in PaWt(Wt-Tg) mice abdominal cavity MNCs had no significant alterations (*p* = 0.153), the proportions of pro-macrophages had an increased tendency in PaWt(Wt-Tg) mice compared with PaWt(Wt-Wt) mice after parabiosis for 8 months (*p* = 0.329) (Fig. 3c-d, g-h).

The in vivo imaging systems (IVIS) spectrum imaging system identified that Aβ_1-42_ peptide labeled with HiLyte Fluor 488 entered the Wt mice circulation after tail intravenous injection 1 h (Fig. 1f). The Aβ_1-42_ plasma levels of Wt mice after injected with Aβ_1-42_ for three times in a week were significantly higher than Wt mice (data not shown). We observed that the proportions of monocytes (both CD11b and CD115 positive), the macrophages (both CD11b and F4/80 positive) in abdominal cavity, the pro-inflammatory macrophages (both CD40 and iNOS positive), and the anti-inflammatory macrophages (both Arg-1 and CD206 positive) had no significant differences between Wt mice and those injected with Aβ_1-42_ for three times in a week (data not shown).

### No effects of the high plasma Aβ_1-42_ on phagocytic ability of macrophages

**Fig. 4:**
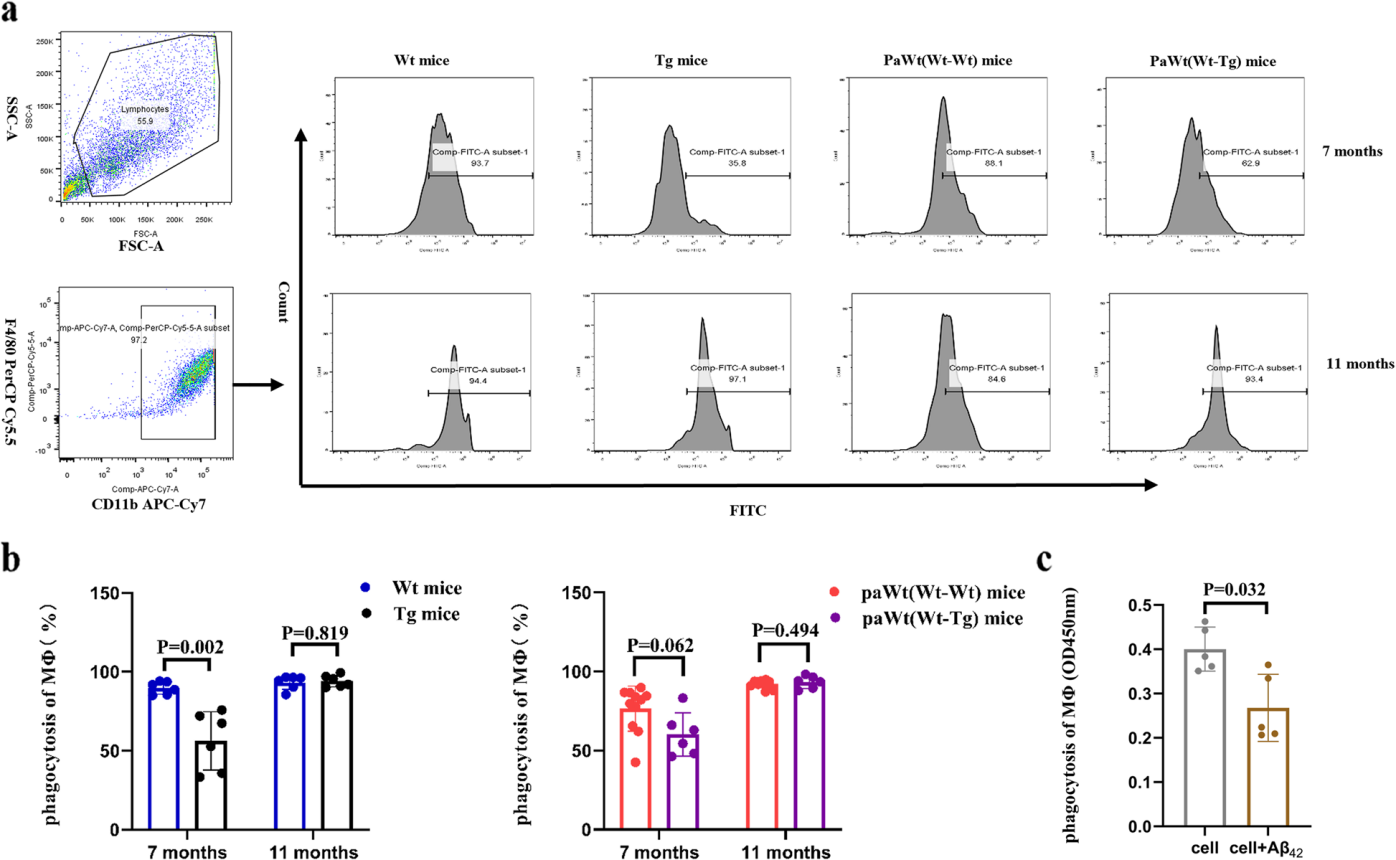
The effects of high plasma Aβ_1-42_ on phagocytic ability of macrophages. **a** The phagocytic ability of macrophages in Wt, Tg, PaWt(Wt-Wt), and PaWt(Wt-Tg) mice. Macrophages were stained with CD11b^+^F4/80.^+^. **b** The statistical diagram of phagocytic ability of macrophages in Wt, Tg, PaWt(Wt-Wt), and PaWt(Wt-Tg) mice. At the age of 7 months, the macrophage phagocytosis Aβ_1-42_ in Tg mice were decreased significantly compared with Wt mice (*p* = 0.002). There is no statistical difference in macrophage phagocytosis Aβ_1-42_ compared Tg with Wt mice at the age of 11 months (*p* = 0.819). After parabiosis for 4 and 8 months, the macrophage phagocytosis Aβ_1-42_ had no obvious alteration (after parabiosis for 4 months *p* = 0.062, after parabiosis for 8 months *p* = 0.494). **c** The phagocytic ability of macrophages after co-cultured with Aβ_1-42_ peptide in vitro. The phagocytosis of macrophages was significantly reduced after co-cultured with Aβ_1-42_ peptide in vitro for 3 days (*p* = 0.032) (**c**). (*n* = 6, Mean ± SD, one-way analysis of variance. *P < 0.05, ns denotes no statistical significance)

As shown in Fig. 4a-b, the macrophage (both CD11b and F4/80 positive) from abdominal cavity were collected, and co-incubated with FITC fluorescent-labeled Aβ_1-42_ peptide for 24 hs. Flow cytometry assays was used to test the phagocytosis of macrophage. At the age of 7 months, the macrophage phagocytosis Aβ_1-42_ in Tg mice were decreased significantly compared with Wt mice (*p* = 0.002). However, there were no statistical difference in macrophage phagocytosis Aβ_1-42_ compared Tg with Wt mice at the age of 11 months (*p* = 0.819) (Fig. 4a-b). After parabiosis for 4 and 8 months, the macrophage phagocytosis Aβ_1-42_ had no obvious alteration (after parabiosis for 4 months *p* = 0.062, after parabiosis for 8 months *p* = 0.494) (Fig. 4a-b). In Wt mice, after tail vein injection with Aβ_1-42_ peptide, the phagocytosis ability of macrophages had no significant change (*p* = 0.690). The macrophages were obtained from Wt mice abdominal cavity. Interestingly, we found that the phagocytosis of macrophages was significantly reduced after co-cultured with Aβ_1-42_ peptide in vitro for 3 days (*p* = 0.032) (Fig. 4c).

### A biphasic regulating effect of the high plasma Aβ_1-42_ on macrophages secreting function

**Fig. 5:**
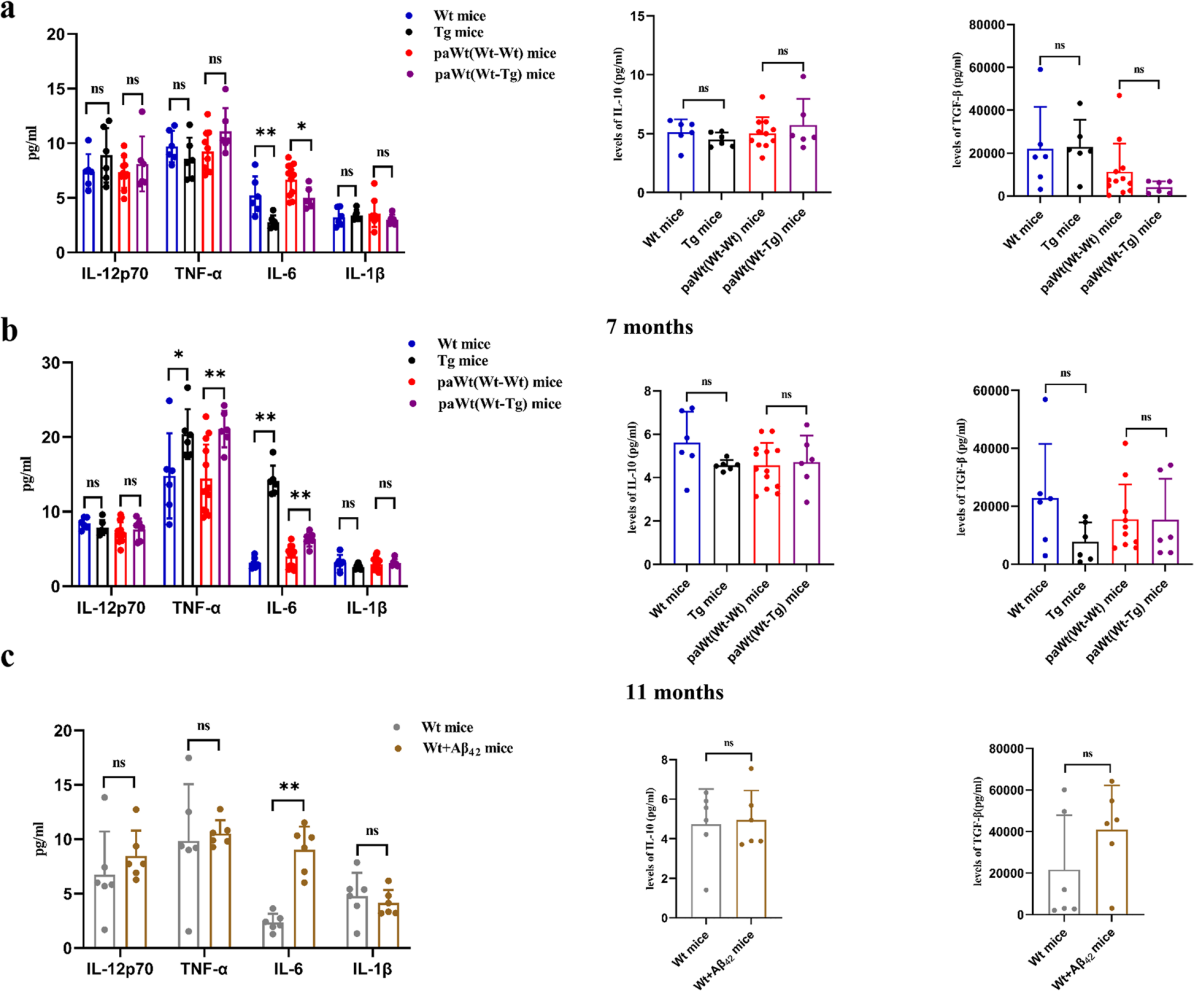
The effects of high plasma Aβ_1-42_ on macrophages secreting function. **a** The plasma levels of cytokines in Wt, Tg, PaWt(Wt-Wt), and PaWt(Wt-Tg) mice at the age of 7 months. At the age of 7 months, the plasma levels of pro-inflammatory cytokines IL-6 in Tg mice were decreased significantly (*p* = 0.04), however, the levels of pro-inflammatory cytokines IL-12p70 (*p* = 0.31), IL-1β (*p* = 0.537) and anti-inflammatory factors such as IL-10 (*p* = 0.244) and TGF-β (*p* = 0.485) in Tg mice had no significant difference compared with Wt mice. Compared with PaWt(Wt-Wt) mice, the plasma levels of IL-6 in PaWt(Wt-Tg) mice were significantly decreased after parabiosis for 4 months (*p* = 0.037), while the levels of pro-inflammatory cytokines IL-12p70 (*p* = 0.95), IL-1β (*p* = 0.25) and anti-inflammatory factors such as IL-10 (*p* = 0.733) and TGF-β (*p* = 0.931) had no significant difference between two groups both after parabiosis for 4 months. **b** The plasma levels of cytokines in Wt, Tg, PaWt(Wt-Wt), and PaWt(Wt-Tg) mice at the age of 11 months. At the age of 11 months, the plasma levels of pro-inflammatory cytokines IL-6 (*p* = 0.002) and TNF-α (*p* = 0.041) were increased significantly compared with Wt mice, however, the levels of pro-inflammatory cytokines IL-12p70 (*p* = 0.310), IL-1β (*p* = 0.132) and anti-inflammatory factors such as IL-10 (*p* = 0.065) and TGF-β (*p* = 0.093) in Tg mice had no significant difference compared with Wt mice. Compared with PaWt(Wt-Wt) mice, after parabiosis for 8 months, the plasma levels of both IL-6 (*p* = 0.002) and TNF-α (*p* = 0.005) were significantly increased in PaWt(Wt-Tg) mice, while the levels of pro-inflammatory cytokines IL-12p70 (*p* = 0.639), IL-1β (*p* = 0.701) and anti-inflammatory factors such as IL-10 (*p* = 0.765) and TGF-β (*p* = 0.525) had no significant difference between PaWt(Wt-Tg) mice and PaWt(Wt-Wt) mice. **c** The plasma levels of cytokines in Wt mice after tail intravenous injection with Aβ_1-42_ peptide and controls injected with PBS. The plasma levels of IL-6 in Wt mice were increased significantly (*p* = 0.000), and the levels of IL-12p70 (*p* = 0.385), TNF-α (*p* = 0.766), IL-10(*p* = 0.827), IL-1β (*p* = 0.542), and TGF-β (*p* = 0.24) exhibited no significant differences after tail intravenous injection with Aβ_1-42_. (*n* = 6 for each group, Mean ± SD, one-way analysis of variance.**P < 0.01, *P < 0.05, ns denotes no statistical significance)

At the age of 7 months, the plasma levels of pro-inflammatory cytokines IL-6 in Tg mice were decreased significantly (*p*=0.04) (Fig. 5a), while at the age of 11 months, the plasma levels of pro-inflammatory cytokines IL-6 (*p*=0.002) and TNF-α (*p*=0.041) were increased significantly compared with Wt mice (Fig. 5b). However, the levels of pro-inflammatory cytokines IL-12p70 (at the age of 7 months *p*=0.310, at the age of 11 months *p*=0.310), IL-1β (at the age of 7 months *p*=0.537, at the age of 11 months *p*=0.132) and anti-inflammatory factors such as IL-10 (at the age of 7 months *p*=0.244, at the age of 11 months *p*=0.065) and TGF-β (at the age of 7 months *p*=0.485, at the age of 11 months *p*=0.093) in Tg mice had no significant difference compared with Wt mice at the age of 7 and 11 months. Compared with PaWt(Wt-Wt) mice, the plasma levels of IL-6 in PaWt(Wt-Tg) mice were significantly decreased after parabiosis for 4 months (*p*=0.037) (Fig. 5a), while after parabiosis for 8 months, the plasma levels of both IL-6 (*p*=0.002) and TNF-α (*p*=0.005) were significantly increased (Fig. 5b). The levels of pro-inflammatory cytokines IL-12p70 (after parabiosis for 4 months *p*=0.953, after parabiosis for 8 months *p*=0.639), IL-1β (after parabiosis for 4 months *p*=0.254, after parabiosis for 8 months *p*=0.701) and anti-inflammatory factors such as IL-10 (after parabiosis for 4 months *p*=0.733, after parabiosis for 8 months *p*=0.765) and TGF-β (after parabiosis for 4 months *p*=0.931, after parabiosis for 8 months *p*=0.525) had no significant difference between two groups both after parabiosis for 4 and 8 months (Fig. 5a-b).

In addition, we also detected the plasma levels of cytokines after tail intravenous injection with Aβ_1-42_. We found the plasma levels of IL-6 in Wt mice were increased significantly (*p* = 0.000), and the levels of IL-12p70 (*p* = 0.385), TNF-α (*p* = 0.766), IL-10 (*p* = 0.827), IL-1β (*p* = 0.542), and TGF-β (*p* = 0.24) exhibited no significant differences after tail intravenous injection with Aβ_1-42_ (Fig. 5c).

### A biphasic regulating effect of the high plasma Aβ_1-42_ on proportions of MDSCs in spleen

**Fig. 6:**
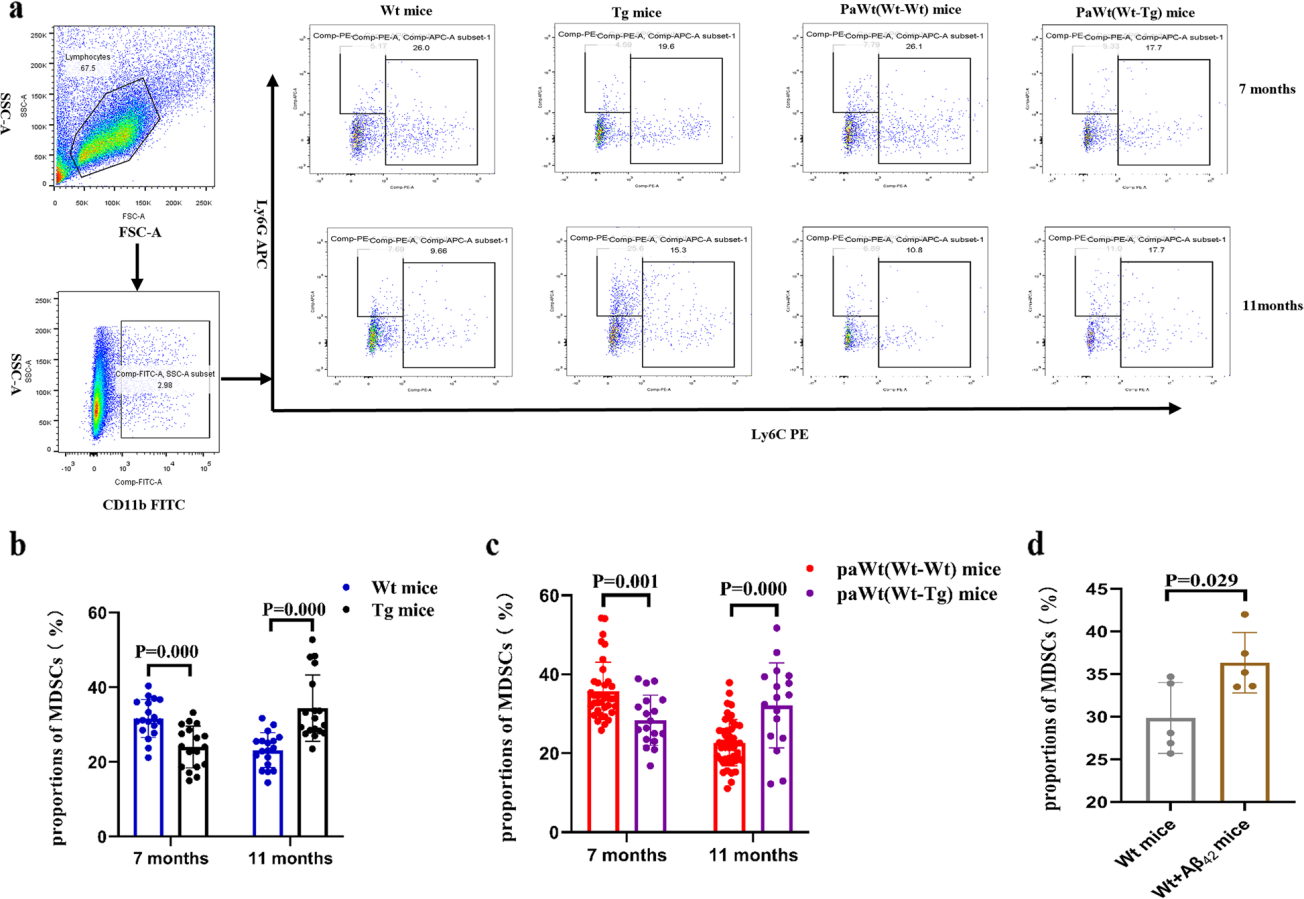
The effects of high plasma Aβ_1-42_ on proportion changes of MDSCs in spleen. **a** The alterations of MDSCs (including PMN-MDSCs and M-MDSCs) proportions in Wt, Tg, PaWt(Wt-Wt), and PaWt(Wt-Tg) mice spleen MNCs. M-MDSCs and PMN-MDSCs were stained with Ly6C^+^Ly6G^−^ and Ly6C^−^Ly6G.^+^, respectively. **b** The statistical diagram of MDSCs in Wt and Tg mice spleen MNCs. At the age of 7 months, the proportions of MDSC in Tg mice spleen MNCs were significantly decreased (*p* = 0.000), while at the age of 11 months, the proportions of MDSCs were increased significantly (*p* = 0.000) when compared with Wt mice. **c** The statistical diagram of MDSCs in PaWt(Wt-Wt), and PaWt(Wt-Tg) mice spleen MNCs. After parabiosis for 4 months, the proportions of MDSCs in PaWt(Wt-Tg) mice spleen MNCs were significantly decreased (*p* = 0.001), while after parabiosis for 8 months, their proportions were significantly increased (*p* = 0.000) compared with PaWt(Wt-Wt) mice. **d** The alterations of MDSCs proportions in Wt mice injected with Aβ_1-42_ and Wt mice. The proportions of MDSCs in Wt mice spleen MNCs were increased significantly after tail intravenous injection with Aβ_1-42_ compared with Wt mice injected with PBS (*p* = 0.029). (Wt, Tg, and PaWt(Wt-Tg) mice *n* = 6 for each group; PaWt(Wt-Wt)mice *n* = 12, data from the two Wt mice of Pa(Wt-Wt) mice, Mean ± SD, one-way analysis of variance, ***P < 0.001, **P < 0.01, ns denotes no statistical significance)

At the age of 7 months, the proportions of MDSCs including PMN-MDSC (both CD11b and Ly6C positive and Ly6G negative) and M-MDSC (both CD11b and Ly6G positive and Ly6C negative) in Tg mice spleen MNCs were significantly decreased (*p*=0.000), while at the age of 11 months, the proportions of MDSCs were increased significantly (*p*=0.000) when compared with Wt mice (Fig. 6a-b). After parabiosis for 4 months, the proportions of MDSCs in PaWt(Wt-Tg) mice spleen MNCs were significantly decreased (*p*=0.001), while after parabiosis for 8 months, their proportions were significantly increased (*p*=0.000) (Fig. 6a, c) compared with PaWt(Wt-Wt) mice. In addition, the proportions of MDSCs in Wt mice spleen MNCs were increased significantly after tail intravenous injection with Aβ_1-42_ compared with Wt mice injected with PBS (*p*=0.029) (Fig. 6d).

### A biphasic regulating effect of the high plasma Aβ_1-42_ on myeloid progenitor cells in bone marrow

**Fig. 7:**
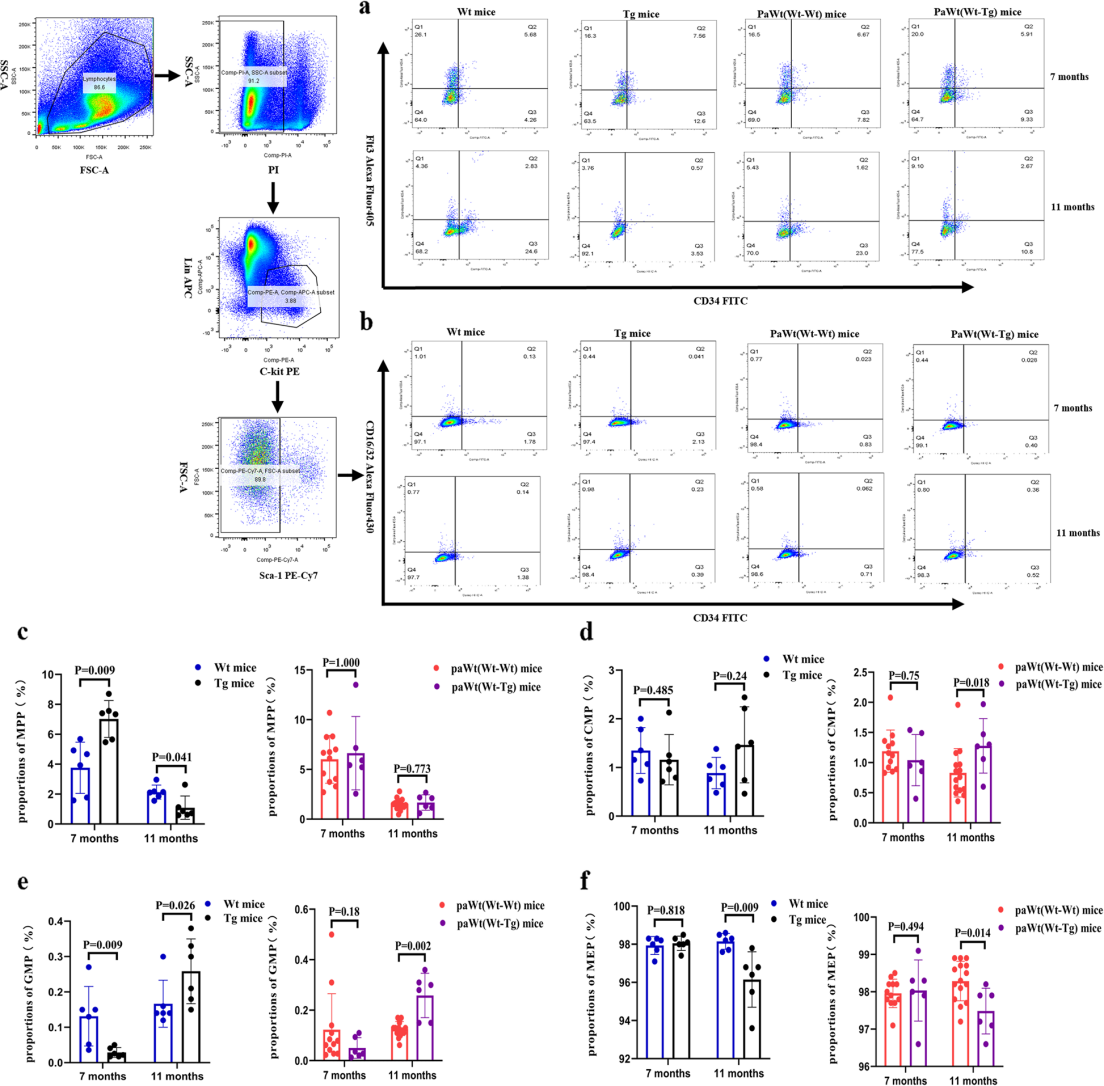
The effects of high plasma Aβ_1-42_ on myeloid progenitor cells in bone marrow. **a** The proportions of MPP in the bone marrow of Wt, Tg, PaWt(Wt-Wt), and PaWt(Wt-Tg) mice. MPP were stained with CD34^+^Flt3^+^. **b** The proportions of CMP, GMP, and MEP in bone marrow of Wt, Tg, PaWt(Wt-Wt), and PaWt(Wt-Tg) mice. CMP, GMP and MEP were stained with CD34^+^CD16/32^−^, CD34^+^CD16/32^+^, and CD34^−^CD16/32.^−^, respectively. **c**-**f** The statistical diagrams of MPP(**c**), CMP(**d**) and GMP(**e**) as well as MEP (**f**) in the bone marrow of Wt, Tg, PaWt(Wt-Wt), and PaWt(Wt-Tg) mice, respectively. Compared Tg mice with Wt mice at 7 months of age, the proportions of MPP increased (*p* = 0.009) and GMP decreased significantly (*p* = 0.009), however both CMP and MEP had no significant changes (CMP p = 0.485, MEP *p* = 0.818). At 11 months of age, in Tg mice, the proportions of MPP and MEP decreased significantly (MPP *p* = 0.041, MEP *p* = 0.009), CMP had no obvious alteration (*p* = 0.24), while GMP increased significantly (*p* = 0.026), when compared with Wt mice. After parabiosis for 4 months, in PaWt(Wt-Tg) mice, the proportions of MPP, CMP, GMP, and MEP had no clear change (MPP *p* = 1.000, CMP *p* = 0.75, GMP *p* = 0.18, MEP *p* = 0.494), after parabiosis for 8 months, CMP and GMP increased significantly (CMP *p* = 0.018, GMP *p* = 0.002), and MEP decreased significantly (*p* = 0.014), however, the proportions of MPP had no visible change (*p* = 0.773), when compared with PaWt(Wt-Wt) mice. (Wt, Tg, PaWt(Wt-Tg) mice *n* = 6; PaWt(Wt-Wt) mice *n* = 12, data from the two Wt mice of Pa(Wt-Wt) mice, Mean ± SD, one-way analysis of variance, **P < 0.01, *P < 0.05, ns denotes no statistical significance)

The proportions of multipotent progenitors (MPPs), common myeloid progenitors (CMPs), granulocyte-monocyte progenitors (GMPs), and megakaryocyte-erythroid progenitors (MEPs) in bone marrow were compared between Tg and Wt mice, PaWt(Wt-Tg) and PaWt(Wt-Wt) mice, respectively. As show in Fig. 7, when compared Tg mice with Wt mice at 7 months of age, the proportions of MPP increased (*p*=0.009) and GMP decreased significantly (*p*=0.009), however both CMP and MEP had no significant changes (CMP *p*=0.485, MEP *p*=0.818), (Fig. 7). At 11 months of age, in Tg mice, the proportions of MPP and MEP decreased significantly (MPP *p*=0.041, MEP *p*=0.009), CMP had no obvious alteration (*p*=0.24), while GMP increased significantly (*p*=0.026), when compared with Wt mice (Fig. 7). After parabiosis for 4 months, in PaWt(Wt-Tg) mice, the proportions of MPP, CMP, GMP, and MEP had no clear change (MPP *p*=1.000, CMP *p*=0.75, GMP *p*=0.18, MEP *p*=0.494) (Fig. 7), and after parabiosis for 8 months, CMP and GMP increased significantly (CMP *p*=0.018, GMP *p*=0.002), and MEP decreased significantly (*p*=0.014), however, the proportions of MPP had no visible change (*p*=0.773), when compared with PaWt(Wt-Wt) mice (Fig. 7). The proportions of MPP, CMP, GMP and MEP in bone marrow of Wt mice had no clear changed after tail vein injection with Aβ_1-42_ (data not shown).

### The high plasma Aβ_1-42_ promoted the proliferation of BMCs

**Fig. 8:**
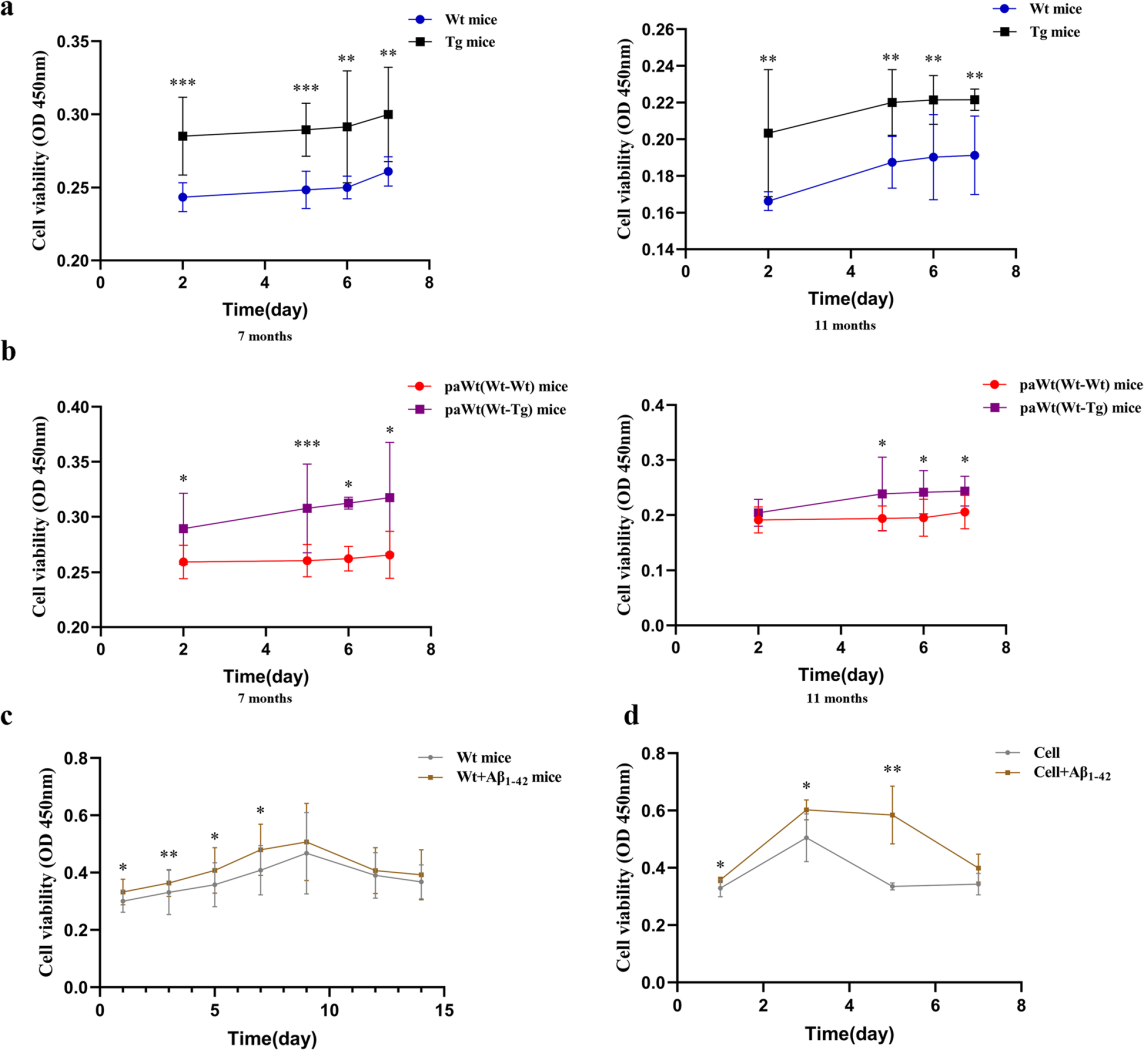
The effects of high plasma Aβ_1-42_ on the proliferation of BMCs. **a** The proliferations of BMCs in Wt and Tg mice at the age of 7 and 11 months. At the ages of 7 months, the proliferations of BMCs in Tg mice were increased significantly compared with Wt mice (at 2, 5 days *p* = 0.000, at 6 days *p* = 0.003, at 7 days *p* = 0.001), the proliferations of BMCs in Tg mice were increased significantly consistently compared with Wt mice at the ages of 11 months (at 2 days *p* = 0.003, at 5 days *p* = 0.001, at 6 and 7 days *p* = 0.009). **b** The proliferations of BMCs in PaWt(Wt-Wt) and paWt(Wt-Tg) mice at the ages of 7 and 11 months. After parabiosis for 4 months, the proliferations of BMCs in PaWt(Wt-Tg) mice were increased significantly compared with PaWt(Wt-Wt) mice (at 2 days *p* = 0.001, at 5 days *p* = 0.000, at 6 and 7 days *p* = 0.001), and the proliferations of BMCs in PaWt(Wt-Tg) mice were increased significantly consistently compared with PaWt(Wt-Wt) mice after parabiosis for 8 months (at 2 days *p* = 0.290, at 5 days *p* = 0.034, at 6 days *p* = 0.006, at 7 days *p* = 0.003). **c** The proliferations of BMCs in Wt mice injected with Aβ_1-42_ and the controls injected with PBS. The proliferations of BMCs in Wt mice were increased significantly after intravenous injection with Aβ_1-42_ (at 1 day *p* = 0.025, at 3 days *p* = 0.004, at 5 days *p* = 0.046, at 7 days *p* = 0.019, at 9 days *p* = 0.371, at 12 days *p* = 0.154, at 14 days *p* = 0.515) compared with those injected with PBS. **d** The proliferations of BMCs after co-cultured with Aβ_1-42_ peptide in vitro. The proliferation of BMCs showed significant increment (at 1 and 3 days *p* = 0.032, at 5 days *p* = 0.008, at 7 days *p* = 0.056) after co-cultured with Aβ_1-42_ peptide in vitro. (*n* = 6, Mean ± SD, one-way analysis of variance, ***P < 0.001, **P < 0.01, *P < 0.05, ns denotes no statistical significance)

The proliferation of BMCs was measured after harvested from mice bone marrow 2, 5, 6, and 7 days respectively in vitro. At the ages of 7 months, the proliferations of BMCs in Tg mice were increased significantly compared with Wt mice (at 2, 5 days *p* = 0.000, at 6 days *p* = 0.003, at 7 days *p* = 0.001), the proliferations of BMCs in Tg mice were increased significantly consistently compared with Wt mice at the ages of 11 months (at 2 days *p* = 0.003, at 5 days *p* = 0.001, at 6 and 7 days *p* = 0.009) (Fig. 8a). After parabiosis for 4 months, the proliferations of BMCs in PaWt(Wt-Tg) mice were increased significantly compared with PaWt(Wt-Wt) mice (at 2 days *p* = 0.001, at 5 days *p* = 0.000, at 6 and 7 days *p* = 0.001), and the proliferations of BMCs in PaWt(Wt-Tg) mice were increased significantly consistently compared with PaWt(Wt-Wt) mice after parabiosis for 8 months (at 2 days *p* = 0.290, at 5 days *p* = 0.034, at 6 days *p* = 0.006, at 7 days *p* = 0.003) (Fig. 8b). The proliferations of BMCs in Wt mice were increased significantly after intravenous injection with Aβ_1-42_ (at 1 day p = 0.025, at 3 days *p* = 0.004, at 5 days *p* = 0.046, at 7 days *p* = 0.019, at 9 days *p* = 0.371, at 12 days *p* = 0.154, at 14 days *p* = 0.515) compared with those injected with PBS (Fig. 8c). Moreover, mice BMCs harvested were co-cultured with Aβ_1-42_ peptide in vitro for 1, 3, 5, and 7 days, respectively, the proliferation of BMCs showed significant increment (at 1 and 3 days *p* = 0.032, at 5 days *p* = 0.008, at 7 days *p* = 0.056) (Fig. 8d).

## Discussion

In the present study, we found that both the plasma Aβ_1-42_ levels and Aβ plaques in PaWt(Wt-Tg) mice were significantly increased similar to Tg mice. Thus, PaWt(Wt-Tg) mice might be a reliable model to investigate the effects and mechanisms of chronic high plasma Aβ_1-42_ levels on mononuclear macrophage, which could eliminate the direct influence of genetic background of Tg mice. Simultaneously, we used PaWt(Wt-Wt) mice as the control group to obviate the effects of parabiotic surgery on PaWt(Wt-Tg) mice.

BMDMs can enter into brain through the damaged BBB and become bone-marrow derived microglia, which are similar to resident microglia in phenotype and function [41]. Nevertheless, the phagocytic capacity of BMDMs is stronger than inherent microglia in mice brain [24, 25].

In our study, BMDM was labeled by IBA-1 bound specifically to microglia and macrophages, and by CD68 with its receptors expressed on activated microglia and macrophages [42] as well as by P2Y12 that was increased in activated microglia and its level distinguishes activated microglia from stationary microglia and BMDMs [43]. The results showed that the amounts of IBA-1^+^CD68^+^ P2Y12^−^ cells did not change obviously after 4 months stimulation with high plasma levels of Aβ_1-42_. However, after 8 months continuous stimulation, these cell amounts were increased significantly, indicating that macrophages could enter into brain through damaged BBB under sustaining Aβ_1-42_ stimulation. Recent studies displayed that Tg mice develop BBB dysfunction that causes varying degrees of barrier leakage at different ages depending on the genetic make-up of the respective model [44, 45]. On the basis of these findings, we speculated that at the early stage of the disease, there was less or lacked of Aβ plaque deposition in mice brain, and the integrity of BBB was not seriously damaged, which was not enough to affect the changes of BMDMs amounts. With the disease aggravation, the deposition of Aβ plaque in mice brain was markedly enhanced and the integrity of BBB may disrupt seriously [46], so the amounts of BMDMs in brain were increased.

Our results showed that after high levels of plasma Aβ_1-42_ stimulation for 4 and 8 months, the proportions of monocytes in the spleen were raised markedly, but the proportions of macrophages in the abdominal cavity had no alteration. Interestingly, after high levels of plasma Aβ_1-42_ stimulation for 4 months, the proportions of pro-inflammatory macrophages in abdominal cavity were decreased obviously, and pro-inflammatory macrophages had an increased tendency after 8 months stimulation. A recent study found that hyperactivation of monocytes and macrophages in mild cognitive impairment (MCI) patients contributes to the AD progression [47]. Monocytes in spleen could clear circulating Aβ and splenectomy might accelerate the development of AD [48]. Under the long-term high plasma levels of Aβ_1-42_ stimulation, monocytes recruit to various damaged sites of the body rapidly, resulting in increasing the proportions of monocytes in spleen [49].

In present study, we found the alterations of pro-inflammatory cytokines and pro-inflammatory macrophages were synchronized. That is, in the early stage of disease, both the amount of pro-inflammatory macrophages and the level of pro-inflammatory cytokines IL-6 were reduced, suggesting that the peripheral immunity of Tg and PaWt(Wt-Tg) mice was in an anti-inflammatory state. In the advanced stage of disease, the amounts of pro-inflammatory macrophages and the levels of pro-inflammatory cytokines TNF-α and IL-6 were enhanced, indicating that the peripheral immunity of Tg and PaWt(Wt-Tg) mice was in a pro-inflammatory state.

In this study, the proportions of monocytes in spleen and macrophages in abdominal cavity had no significant alteration after injected with Aβ_1-42_ for three times in a week. Since AD and its animal models is a chronic progressive degenerative disease, acute short time Aβ_1-42_ stimulation did not affect the proportions of mononuclear macrophages.

Until now, the results of phagocytosis ability of macrophage on Aβ_1-42_ were controversial. The phagocytic capacity of all three subsets of monocytes, including non-classic, intermediate and classic monocytes, were similar between healthy controls and AD patients, while the basal phagocytosis of monocytes in subjects with high Aβ-amyloid burden was increased significantly. Moreover, pre-treating three subsets of monocytes with copaxone (a stimulator of phagocytosis) or ATP (an inhibitor of phagocytosis), showed a differential response depending on clinical or Aβ-burden status, so it indicated the phagocytic function of monocytes was relatively deficient in AD patients [50]. In another study, the uptake of Aβ_1-42_ by monocytes was decreased during ageing and was further decreased in AD patients. Moreover, Aβ_1-42_ uptake by monocytes was negatively correlated with Aβ_1-42_ levels in blood [51]. Although high plasma Aβ_1-42_ continue stimulation could increase the proportions of peripheral monocytes, the expressions of pro-inflammatory cytokines IL-6 and TNF-α, and the infiltrations of BMDMs into brain, no increases in phagocytosis of Aβ_1-42_ by macrophages was observed in our study. Collectively, we think that it might be unreasonable to activate peripheral mononuclear macrophage into brain parenchyma and to replace the ageing microglia of AD.

**Fig. 9:**
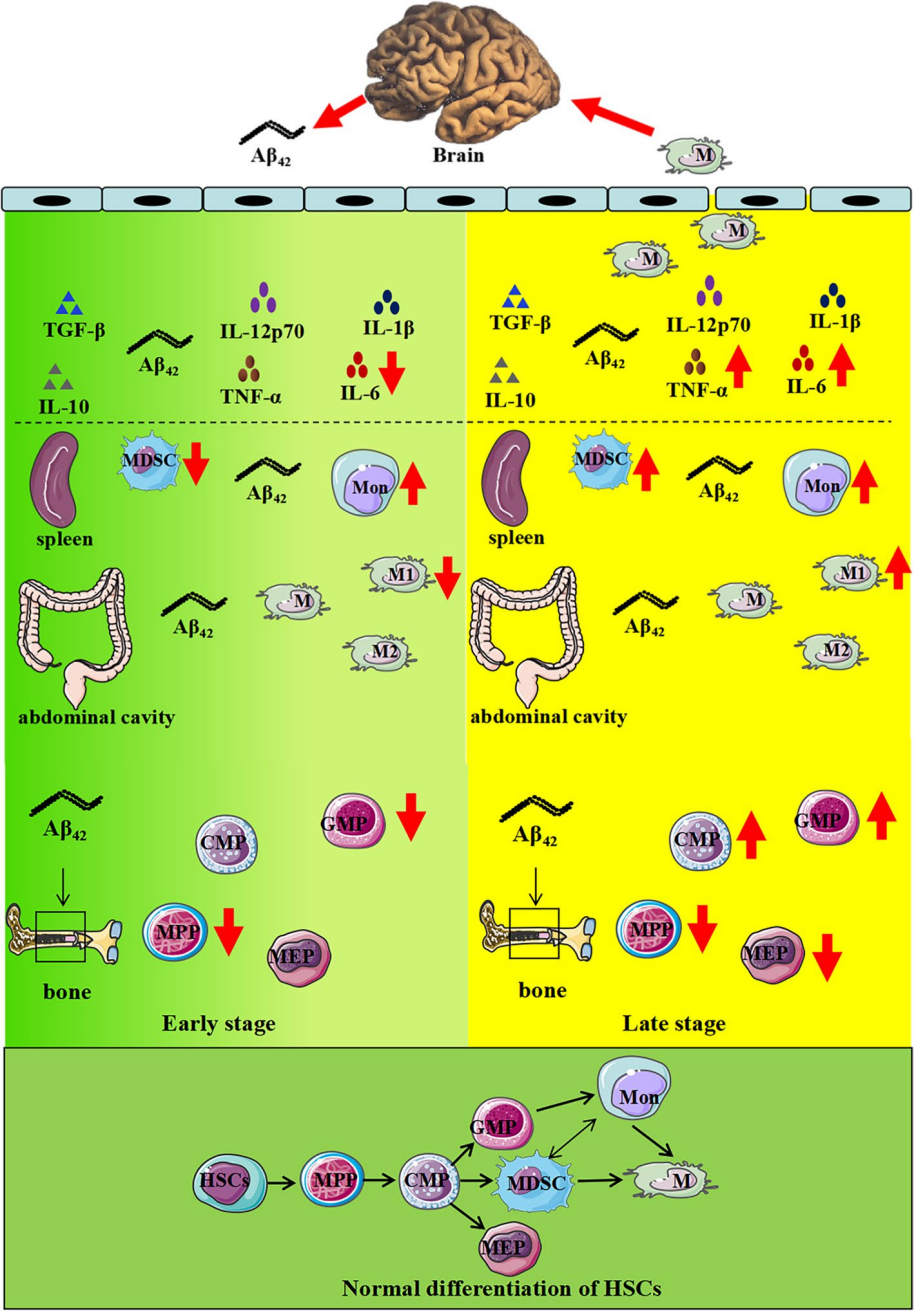
Schematic diagram depicting of the effects of high plasma Aβ_1-42_ levels on peripheral innate immune cells and HSCs. In early stage (7-month-old) of AD mouse models, high plasma Aβ_1-42_ levels reduced the amounts of pro-inflammatory macrophages, MDSCs, GMP, as well as the plasma levels of pro-inflammatory cytokines significantly. In late stage (11-month-old), high plasma Aβ_1-42_ levels increased the amounts of pro-inflammatory macrophages, MDSCs, GMP, and plasma levels of pro-inflammatory cytokines, as well as infiltrations of BMDMs in mice brain. In addition, high plasma Aβ_1-42_ levels increased the amounts of peripheral monocytes significantly

We further investigated the mechanisms of high plasma levels of Aβ_1-42_ affecting mononuclear macrophage. To the best of our knowledge, this is the first time to study the effects of high plasma levels of Aβ_1-42_ on the proliferation of BMCs. In our study, the proliferation of BMCs was clear increased after high plasma levels of Aβ_1-42_ stimulation. The proportions of MDSCs in spleen, and GMP (the precursor cells of monocytes and macrophages in the plasma) in bone marrow were obviously decreased after stimulation with high plasma levels of Aβ_1-42_ for 4 months, which are consistent with the alterations of pro-inflammatory macrophages in abdominal cavity. MDSCs are the most important immune modulatory cells of innate immune system. In fact, the role of MDSCs is still unclear. In the early stages of chronic inflammation, immature BMCs with MDSC-like phenotype may not have effective immunosuppressive activity [52]. Thus, MDSCs do not play an immunosuppressive role in the early stage of AD, while with the progression of the disease, under the continuous effects of chronic inflammation, MDSCs induced the expressions of Arg-1 and indoleamine-pyrrole 2,3-dioxygenase (IDO) 1 by secreting anti-inflammatory factors IL-10 and TGF-β, inhibited the proliferations of inflammatory cells and exerted an immunosuppressive efficacy [53]. On contrary to our findings, a previous study reported that the amounts of MDSCs were significantly increased in the peripheral blood of amnesic MCI patients and decreased in mild AD patients [54]. In AD, MDSCs maintain the immunosuppressive microenvironment and reduce the direct damage to neurons, but on the other hand, MDSCs may increase the depositions of Aβ and the amounts of cell fragments in brain, aggravating the pathological changes of AD. So far, there is no direct evidence that MDSCs can enter into AD brain. Analysis of MDSCs in brain is more difficult because cell sorting techniques are not applicable to postmortem brains and freezing results in a significant reduction in granulocytic MDSCs (G-MDSCs). Therefore, the study of MDSCs in AD faces more challenges. Aβ and Tau were found to deposit in bone marrow, and Aβ promotes osteogenic differentiation via Wnt/β-catenin signaling and inhibits osteoclast differentiation via OPG/RANKL/RANK system. In addition, the activation of osteoclast was regulated by Aβ in an age-dependent manner [55–57]. To date, there are very few studies about the changes of hematopoietic stem cells (HSCs) in AD patients and its animal models. A previous study reported the proportions of short-term HSC in 12 months-old 3 × Tg mice were decreased significantly [58]. The proportions of CMP and GMP in bone marrow were decreased after 4 months continuous stimulation with high plasma levels of Aβ_1-42_, and increased significantly after 8 months continuous stimulation, which is consistent with the alterations of MDSCs and macrophages in the peripheral circulation. CMP in bone marrow can be differentiated into GMP and MDSCs, and GMP eventually differentiates into mononuclear macrophage and MDSCs in peripheral circulation. AD patients are often accompanied with anemia, suggesting there is a significant correlation between anemia and AD [59]. In our study, the proportions of MEP in the bone marrow decreased significantly after long-term stimulation with high plasma levels of Aβ_1-42_, which supported that Aβ can lead to anemia by influencing the amounts of MEP in erythrocyte progenitors. In atherosclerosis, bone marrow mesenchymal stem cells increased production of cytokines, such as TNF-α, IL-6, and IL-1, which regulated the differentiation of HSCs via affecting the hematopoietic microenvironment of bone marrow [60]. In our study, the differentiation of HSCs was affected by the alterations of bone marrow hematopoietic microenvironment under the continuous stimulation of Aβ_1-42_. In AD, genes, bone marrow microenvironment and peripheral immune cells may be involved in regulating the differentiation of HSCs, which in turn affects peripheral immune cells. Therefore, we suggest that the alterations of the amounts and proliferations of myeloid progenitor cells in bone marrow may be involved in the regulation of peripheral MDSCs and mononuclear macrophage, intervening the effects of plasma Aβ_1-42_ on mononuclear macrophage may be a new therapeutic strategy to AD (Fig. 9).

There are some limitations in our study. Firstly, the cell signal pathways of plasma Aβ_1-42_ on monocytes, macrophages, MDSCs and HSCs remains unclear. Secondly, we did not study the effects of plasma Aβ_1-42_ on the subtype of splenic monocytes, including non-classic (CD14dimCD16 +) monocytes, intermediate (CD14 + CD16 +) monocytes and classic (CD14 + CD16-) monocytes. Lastly, we also did not study the effects of plasma Aβ_1-42_ on the amounts and functions of monocytes, macrophages, MDSCs and HSCs in the stages of preclinical, prodromal and clinical AD patients.

## Conclusion

Applying the established the parabiosis model of Wt mice and APP/PS1 Tg mice investigating the effects of high plasma levels of Aβ_1-42_ on peripheral mononuclear macrophage, which closely resembles dominantly inherited AD and Down syndrome, may furthermore, be a useful tool for study of AD pathogenesis. In the early stage of disease, high plasma levels of Aβ_1-42_ may inhibit the activation of peripheral inflammatory processes, whereas in the late stage, high plasma levels of Aβ_1-42_ may over-activate the peripheral and central inflammatory processes by affecting the proliferation and differentiation of HSCs. Further studies are needed to explore the mechanism behind the biphasic regulating effect of the high plasma Aβ_1-42_ on mononuclear macrophage and to determine whether intervention these effects is a new therapeutic strategy to AD.

## Data Availability

The data that support the findings of this study are available on request from the corresponding author. The data are not publicly available due to privacy or ethical restrictions.

## References

[1] Scheltens P, De Strooper B, Kivipelto M (2021). Alzheimer's disease. Lancet.

[2] Jia J, Wei C, Chen S (2018). The cost of Alzheimer's disease in China and re-estimation of costs worldwide. Alzheimers Dement.

[3] GBD 2016 Dementia Collaborators (2019). Global, regional, and national burden of Alzheimer's disease and other dementias, 1990–2016: a systematic analysis for the Global Burden of Disease Study 2016. Lancet Neurol..

[4] 2020 Alzheimer's disease facts and figures. Alzheimers Dement. 2020. 10.1002/alz.12068. 10.1002/alz.12068 32157811

[5] Long JM, Holtzman DM (2019). Alzheimer disease: an update on pathobiology and treatment strategies. Cell.

[6] Gauthier S, Feldman HH, Schneider LS (2016). Efficacy and safety of tau-aggregation inhibitor therapy in patients with mild or moderate Alzheimer's disease: a randomised, controlled, double-blind, parallel-arm, phase 3 trial. Lancet.

[7] Alexander GC, Emerson S, Kesselheim AS (2021). Evaluation of Aducanumab for Alzheimer disease: scientific evidence and regulatory review involving efficacy, safety, and futility. JAMA.

[8] Harrison JR, Owen MJ (2016). Alzheimer's disease: the amyloid hypothesis on trial. Br J Psychiatry.

[9] Kametani F, Hasegawa M (2018). Reconsideration of amyloid hypothesis and tau hypothesis in Alzheimer's disease. Front Neurosci.

[10] Makin S (2018). The amyloid hypothesis on trial. Nature.

[11] Tolar M, Abushakra S, Sabbagh M (2020). The path forward in Alzheimer's disease therapeutics: reevaluating the amyloid cascade hypothesis. Alzheimers Dement.

[12] Heneka MT, Carson MJ, El Khoury J (2015). Neuroinflammation in Alzheimer's disease. Lancet Neurol.

[13] Nimmerjahn A, Kirchhoff F, Helmchen F (2005). Resting microglial cells are highly dynamic surveillants of brain parenchyma in vivo. Science.

[14] Hickman SE, Allison EK, El Khoury J (2008). Microglial dysfunction and defective beta-amyloid clearance pathways in aging Alzheimer's disease mice. J Neurosci.

[15] Sims R, van der Lee SJ, Naj AC (2017). Rare coding variants in PLCG2, ABI3, and TREM2 implicate microglial-mediated innate immunity in Alzheimer's disease. Nat Genet.

[16] Kunkle BW, Grenier-Boley B, Sims R (2019). Genetic meta-analysis of diagnosed Alzheimer's disease identifies new risk loci and implicates Aβ, tau, immunity and lipid processing. Nat Genet.

[17] Hong S, Beja-Glasser VF, Nfonoyim BM (2016). Complement and microglia mediate early synapse loss in Alzheimer mouse models. Science.

[18] Dani M, Wood M, Mizoguchi R (2018). Microglial activation correlates in vivo with both tau and amyloid in Alzheimer's disease. Brain.

[19] Pascoal TA, Benedet AL, Ashton NJ (2021). Microglial activation and tau propagate jointly across Braak stages. Nat Med.

[20] Wang J, Gu BJ, Masters CL, Wang YJ (2017). A systemic view of Alzheimer disease - insights from amyloid-β metabolism beyond the brain. Nat Rev Neurol.

[21] Prinz M, Priller J (2017). The role of peripheral immune cells in the CNS in steady state and disease. Nat Neurosci.

[22] Zhu F, Li C, Chu F, Tian X, Zhu J (2020). Target Dysbiosis of gut microbes as a future therapeutic manipulation in Alzheimer's disease. Front Aging Neurosci.

[23] Shi M, Chu F, Tian X, Aerqin Q, Zhu F, Zhu J. Role of adaptive immune and impacts of risk factors on adaptive immune in alzheimer's disease: are immunotherapies effective or off-target? Neuroscientist. 2022;28(3):254–70. 10.1177/1073858420987224 33530843

[24] Simard AR, Soulet D, Gowing G, Julien JP, Rivest S (2006). Bone marrow-derived microglia play a critical role in restricting senile plaque formation in Alzheimer's disease. Neuron.

[25] Kawanishi S, Takata K, Itezono S (2018). Bone-marrow-derived microglia-like cells ameliorate brain amyloid pathology and cognitive impairment in a mouse model of Alzheimer's disease. J Alzheimers Dis.

[26] Butovsky O, Kunis G, Koronyo-Hamaoui M, Schwartz M (2007). Selective ablation of bone marrow-derived dendritic cells increases amyloid plaques in a mouse Alzheimer's disease model. Eur J Neurosci.

[27] Koronyo Y, Salumbides BC, Sheyn J (2015). Therapeutic effects of glatiramer acetate and grafted CD115^+^ monocytes in a mouse model of Alzheimer's disease. Brain.

[28] Magga J, Savchenko E, Malm T (2012). Production of monocytic cells from bone marrow stem cells: therapeutic usage in Alzheimer's disease. J Cell Mol Med.

[29] Stewart WF, Kawas C, Corrada M, Metter EJ (1997). Risk of Alzheimer's disease and duration of NSAID use. Neurology.

[30] Yip AG, Green RC, Huyck M, Cupples LA, Farrer LA, MIRAGE Study Group (2005). Nonsteroidal anti-inflammatory drug use and Alzheimer's disease risk: the MIRAGE Study. Bmc Geriatr.

[31] Etminan M, Gill S, Samii A (2003). Effect of non-steroidal anti-inflammatory drugs on risk of Alzheimer's disease: systematic review and meta-analysis of observational studies. BMJ.

[32] Wang X, Sun G, Feng T, Zhang J, Huang X, Wang T (2019). Sodium oligomannate therapeutically remodels gut microbiota and suppresses gut bacterial amino acids-shaped neuroinflammation to inhibit Alzheimer's disease progression. Cell Res.

[33] Zenaro E, Pietronigro E, Della Bianca V (2015). Neutrophils promote Alzheimer's disease-like pathology and cognitive decline via LFA-1 integrin. Nat Med.

[34] Teunissen CE, Verberk I, Thijssen EH (2022). Blood-based biomarkers for Alzheimer's disease: towards clinical implementation. Lancet Neurol.

[35] Bateman RJ, Xiong C, Benzinger TL (2012). Clinical and biomarker changes in dominantly inherited Alzheimer's disease. N Engl J Med.

[36] Fortea J, Vilaplana E, Carmona-Iragui M (2020). Clinical and biomarker changes of Alzheimer's disease in adults with Down syndrome: a cross-sectional study. Lancet.

[37] Jia L, Qiu Q, Zhang H (2019). Concordance between the assessment of Aβ42, T-tau, and P-T181-tau in peripheral blood neuronal-derived exosomes and cerebrospinal fluid. Alzheimers Dement.

[38] Bu XL, Xiang Y, Jin WS (2018). Blood-derived amyloid-β protein induces Alzheimer's disease pathologies. Mol Psychiatry.

[39] Sun HL, Chen SH, Yu ZY (2021). Blood cell-produced amyloid-β induces cerebral Alzheimer-type pathologies and behavioral deficits. Mol Psychiatry.

[40] Yang C, Liu ZL, Wang J, Bu XL, Wang YJ, Xiang Y (2021). Parabiosis modeling: protocol, application and perspectives. Zool Res..

[41] Mammana S, Fagone P, Cavalli E, Basile MS, Petralia MS, Nicoletti F, Bramanti P, Mazzon E (2018). The Role of Macrophages in Neuroinflammatory and Neurodegenerative Pathways of Alzheimer's Disease, Amyotrophic Lateral Sclerosis, and Multiple Sclerosis: Pathogenetic Cellular Effectors and Potential Therapeutic Targets. Int J Mol Sci..

[42] Stankov A, Belakaposka-Srpanova V, Bitoljanu N, Cakar L, Cakar Z, Rosoklija G (2015). Visualisation of microglia with the use of immunohistochemical double staining method for CD-68 and Iba-1 of cerebral tissue samples in cases of brain contusions. Pril (Makedon Akad Nauk Umet Odd Med Nauki).

[43] Mildner A, Huang H, Radke J, Stenzel W, Priller J (2017). P2Y receptor is expressed on human microglia under physiological conditions throughout development and is sensitive to neuroinflammatory diseases. Glia.

[44] Nehra G, Bauer B, Hartz AMS (2022). Blood-brain barrier leakage in Alzheimer's disease: From discovery to clinical relevance. Pharmacol Ther..

[45] Sweeney MD, Sagare AP, Zlokovic BV (2018). Blood-brain barrier breakdown in Alzheimer disease and other neurodegenerative disorders. Nat Rev Neurol.

[46] Montagne A, Barnes SR, Sweeney MD, Halliday MR, Sagare AP (2015). Blood-brain barrier breakdown in the aging human hippocampus. Neuron..

[47] Munawara U, Catanzaro M, Xu W (2021). Hyperactivation of monocytes and macrophages in MCI patients contributes to the progression of Alzheimer's disease. Immun Ageing.

[48] Yu ZY, Chen DW, Tan CR (2022). Physiological clearance of Aβ by spleen and splenectomy aggravates Alzheimer-type pathogenesis. Aging Cell.

[49] Shi C, Pamer EG (2011). Monocyte recruitment during infection and inflammation. Nat Rev Immunol.

[50] Gu BJ, Huang X, Ou A (2016). Innate phagocytosis by peripheral blood monocytes is altered in Alzheimer's disease. Acta Neuropathol.

[51] Chen SH, Tian DY, Shen YY (2020). Amyloid-beta uptake by blood monocytes is reduced with ageing and Alzheimer's disease. Transl Psychiatry.

[52] Salminen A, Kaarniranta K, Kauppinen A (2018). The role of myeloid-derived suppressor cells (MDSC) in the inflammaging process. Ageing Res Rev.

[53] Salminen A, Kaarniranta K, Kauppinen A (2018). The potential importance of myeloid-derived suppressor cells (MDSCs) in the pathogenesis of Alzheimer's disease. Cell Mol Life Sci CMLS.

[54] Le Page A, Garneau H, Dupuis G (2017). Differential phenotypes of myeloid-derived suppressor and T regulatory cells and cytokine levels in amnestic mild cognitive impairment subjects compared to mild Alzheimer diseased patients. Front Immunol.

[55] Li S, Liu B, Zhang L, Rong L (2014). Amyloid beta peptide is elevated in osteoporotic bone tissues and enhances osteoclast function. Bone.

[56] Margulies BS, DeBoyace SD, Parsons AM, Policastro CG, Ee JS, Damron TS (2015). Functionally deficient mesenchymal stem cells reside in the bone marrow niche with M2-macrophages and amyloid-β protein adjacent to loose total joint implants. J Orthop Res.

[57] Yang B, Li S, Chen Z (2020). Amyloid β peptide promotes bone formation by regulating Wnt/β-catenin signaling and the OPG/RANKL/RANK system. FASEB J.

[58] St-Amour I, Bosoi CR, Paré I (2019). Peripheral adaptive immunity of the triple transgenic mouse model of Alzheimer's disease. J Neuroinflammation.

[59] Ellis K. A, Rumble, R. L, Martins, R. N, Wiley, Ames, Bush, A. I: An anemia of Alzheimer's disease. Mol Psychiatry. 2014;19(11):1227–34. 10.1038/mp.2013.178 24419041

[60] Cortez M, Carmo LS, Rogero MM (2013). A high-fat diet increases IL-1, IL-6, and TNF-alpha production by increasing NF-kappa B and attenuating PPAR-gamma expression in bone marrow mesenchymal stem cells. Inflammation.

